# Reproductive health among married and unmarried mothers aged less than 18, 18–19, and 20–24 years in the United States, 2014–2019: A population-based cross-sectional study

**DOI:** 10.1371/journal.pmed.1003929

**Published:** 2022-03-10

**Authors:** Andrée-Anne Fafard St-Germain, Russell S. Kirby, Marcelo L. Urquia

**Affiliations:** 1 Manitoba Centre for Health Policy, Community Health Sciences, Max Rady College of Medicine, Rady Faculty of Health Sciences, University of Manitoba, Winnipeg, Manitoba, Canada; 2 Chiles Center, College of Public Health, University of South Florida, Tampa, Florida, United States of America; 3 Dalla Lana School of Public Health, University of Toronto, Toronto, Ontario, Canada; London School of Hygiene and Tropical Medicine, UNITED KINGDOM

## Abstract

**Background:**

Studies in low- and middle-income regions suggest that child marriage (<18 years) is a risk factor for poor reproductive outcomes among women. However, in high-income-country contexts where childbearing before age 18 occurs predominantly outside marriage, it is unknown whether marriage is adversely associated with reproductive health among mothers below age 18. This study examined the joint associations of marriage and adolescent maternal age group (<18, 18–19, and 20–24 years) with reproductive, maternal, and infant health indicators in the United States.

**Methods and findings:**

Birth registrations with US resident mothers aged ≤24 years with complete information on marital status were drawn from the 2014 to 2019 Natality Public Use Files (*n* = 5,669,824). Odds ratios for the interaction between marital status and maternal age group were estimated using multivariable logistic regression, adjusting for covariates such as maternal race/ethnicity and nativity status, federal program participation, and paternal age. Marriage prevalence was 3.6%, 13.2%, and 34.1% among births to mothers aged <18, 18–19, and 20–24 years, respectively. Age gradients in the adjusted odds ratios (AORs) were present for most indicators, and many gradients differed by marital status. Among births to mothers aged <18 years, marriage was associated with greater adjusted odds of prior pregnancy termination (AOR 1.64, 95% CI 1.52–1.77, *p <* 0.001), repeat birth (AOR 2.84, 95% CI 2.68–3.00, *p <* 0.001), maternal smoking (AOR 1.24, 95% CI 1.15–1.35, *p <* 0.001), and infant morbidity (AOR 1.07, 95% CI 1.01–1.14, *p* = 0.03), but weaker or reverse associations existed among births to older mothers. For all maternal age groups, marriage was associated with lower adjusted odds of late or no prenatal care initiation, sexually transmitted infection, and no breastfeeding at hospital discharge, but these beneficial associations were weaker among births to mothers aged <18 and 18–19 years. Limitations of the study include its cross-sectional nature and lack of information on marriage timing relative to prior pregnancy events.

**Conclusions:**

Marriage among mothers below age 18 is associated with both adverse and favorable reproductive, maternal, and infant health indicators. Heterogeneity exists in the relationship between marriage and reproductive health across adolescent maternal age groups, suggesting girl child marriages must be examined separately from marriages at older ages.

## Abstract 2

In a population-based study, Andrée-Anne Fafard St-Germain and colleagues examine the joint associations of marriage and adolescent maternal age group (<18, 18-19, and 20-24 years) with reproductive, maternal, and infant health indicators in the United States.

## Author summary

**Why was this study done?:**

- Research in low- and middle-income countries indicates that child marriage, namely marriage before age 18 years, has negative implications for the reproductive health and well-being of women compared to marriage at an older age.
- In high-income countries where childbearing increasingly occurs outside marriage, it is unclear how marriage among mothers below age 18 relates to different indicators of reproductive health.
- Studies in high-income countries show that marriage is associated with positive reproductive health outcomes, but it is unknown whether these beneficial associations apply to mothers below age 18.

**What did the researchers do and find?:**

- We analyzed cross-sectional information from more than 5.5 million birth registrations with mothers aged ≤24 years in the United States from 2014 to 2019 to examine how marriage and maternal age jointly relate to reproductive health.
- Marriage was associated with increased odds of prior pregnancy termination, repeat childbearing, maternal smoking, and infant morbidity among births to mothers below age 18, but the association of marriage with these indicators was weaker or reversed among births to older mothers.
- Protective associations with marriage were observed for preterm birth, small-for-gestational-age birth, sexually transmitted infection, prenatal care initiation, and breastfeeding among births to mothers aged 20–24 years, but for births to mothers aged <18 and 18–19 years, these beneficial associations were weaker or absent.

**What do these findings mean?:**

- Our study indicates that the health advantage of marriage observed among mothers aged 20–24 years in the United States may not consistently apply to younger mothers.
- It suggests that the association between marriage below age 18 and reproductive health indicators is context- and outcome-dependent.
- Although this study cannot establish causation, it suggests the need for further research on the driving forces and the health and social consequences of marriage before age 18 in the US and other high-income countries, and that such research may benefit from longitudinal study designs.

## Introduction

According to international conventions, child marriage, the marriage or union of an individual below age 18 years, poses serious human rights concerns, including potential violations of the rights of the child to protection from abuses and harmful traditional practices [1]; the right to informed, free, and full consent to marriage [2]; and women’s rights to non-discrimination [3,4]. More girls than boys marry before age 18 [4,5], and ending child marriage is part of the international agenda to achieve the Sustainable Development Goal of gender equality by 2030 [6]. Globally, approximately 12 million girls marry before turning 18 annually [4]. Although the marriage of girls is most prevalent in sub-Saharan Africa and South Asia [4], it occurs worldwide, including the United States (US) [5,7].

While the minimum legal age to marry in the US is generally 18 years, in all except 4 states, statutory exceptions permit marriage before age 18 if the minor is emancipated (legally considered an adult) or if there is parental consent, judicial approval, and/or a pregnancy [7,8]. Most states permitting child marriage have statutory age floors between 14 and 17 years, below which marriage is fully proscribed, but many still have no minimum age requirement [8]. The US immigration system also authorizes adult citizens to file a visa petition for a minor spouse if the marriage was lawful in the country of occurrence and is considered valid in the state of residence [9,10]. A recent population-based study in the US estimated that 7 in 1,000 girls and 6 in 1,000 boys aged 15 to 17 years had been or were married in 2010–2014, with rates varying by racial and ethnic background, birthplace, and state of residence [5].

Studies from low- and middle-income countries (LMICs) indicate that women who marry before age 18 are more likely to achieve lower educational attainment [11–13] and to experience limited autonomy [13], intimate partner violence [14], unintended pregnancies [13,15,16], pregnancy termination [13,15–18], and higher lifetime fertility [12,13,15,18] than those who marry at an older age. As child marriage often coincides with early childbearing in these countries, it may also indirectly contribute to poor maternal and infant outcomes [19–22]. Given that the meanings of both “child” and “marriage” are socially constructed and context dependent [23], it is uncertain whether findings from LMICs are generalizable to the US. The relationships documented between child marriage and reproductive health in LMICs focus on the comparison of women who marry before age 18 to those who marry after that age in country contexts where childbearing predominantly occurs within marriage [12,13,15–18]. In the US, extramarital childbearing has been common for several decades [24–26], and most US mothers giving birth before age 18 are now unmarried [25,26].

Studies from high-income-country contexts predominantly show that at the population level, married mothers are less likely to experience intimate partner violence [27], report substance use during pregnancy [27,28], and have poor birth outcomes [29,30] than mothers who are single or in a common-law union. These findings indicate that married mothers below age 18 may have better outcomes than those who are unmarried, but it is unclear if the beneficial associations with marriage among adult mothers [27,29,30] extend to those below age 18, as they are developmentally different [31]. To date, it remains unknown how marriage relates to reproductive health among mothers below age 18 in the US.

We utilized national birth records from 2014 to 2019 to examine the joint associations of marriage and adolescent maternal age group (<18, 18–19, and 20–24 years) with different indicators of reproductive, maternal, and infant health in the US. Given the paucity of data on reproductive health and child marriages in the US [5,7], our study focused on the interplay between marital status and maternal age group among recent live births to shed light on the contemporary health implications of child marriage in this country. In testing for the interaction between marital status and adolescent maternal age group, the study assessed whether associations between marriage and reproductive, maternal, and infant health indicators differ between births to mothers aged <18, 18–19, and 20–24 years. Considering the well-documented age differences in the risk of pregnancy and infant outcomes among adolescent mothers [19–22], the study also assessed, via the interaction, if the age gradients in the odds of the indicators vary between births to married and unmarried mothers.

## Methods

### Data source

The data were derived from the 2014 to 2019 Natality Public Use Files (Birth Data Files) provided by the National Center for Health Statistics [32]. These annual files contain information on parental demographic characteristics, maternal medical conditions, and pregnancy outcomes for all live births registered in the 50 states, District of Columbia (DC), and US territories. Data were collected by hospital staff via maternal self-reporting and the medical records of the mother and newborn [33–38]. The 2003 US Standard Certificate of Live Birth used to standardize data collection was partially or fully implemented in 49 states and DC in 2014 and 2015, representing more than 96% of births to US residents, and was nationally implemented in 2016 [33–35]. The data files for 2014 and 2015 were included in the study to maximize sample size given the low prevalence of child marriage documented in the US [5] and the high proportion of births recorded using the 2003 birth certificate during those years [33,34].

### Analysis plan

The analysis plan was developed from January to June 2020 prior to data analyses, but no written protocol was created. The reproductive, maternal, and infant health indicators and the covariates were selected a priori after careful review of the user guides for the 2014 to 2018 Natality Public Use Files [33–37], the international literature on child marriage [5,12,13,15–18], and research on teen pregnancy [19–22,39]. The original analyses were conducted from June 2020 to December 2020 using the 2014 to 2018 Natality Public Use Files, which were the most recent data files available at the start of the analyses; the 2019 data file was appended to the study in August 2021 given its availability at the time of peer review. Several sensitivity analyses were also conducted in August 2021 in response to peer review to test the robustness of the findings. This study is reported following the Strengthening the Reporting of Observational Studies in Epidemiology (STROBE) Guideline (S1 Checklist). As a secondary data analysis study relying on publicly available data, ethical approval from the data provider or the authors’ institutions was not required to conduct the study.

### Study sample

There were 23,366,890 live births registered between 1 January 2014 and 31 December 2019. The study sample included 5,669,824 births to US resident mothers aged ≤24 years with complete information on marital status and recorded using the 2003 birth certificate. For the analysis of most maternal and infant health indicators, the sample was further limited to 5,532,654 singleton births with gestational age ≥ 20 weeks and birth weight ≥ 350 g [40]. For the indicator small for gestational age (SGA), the sample was restricted to 5,522,736 births with gestational age between 24 and 42 weeks due to the gestational age limits of the INTERGROWTH-21st newborn birth weight charts [41]. Birth records with a missing value for an indicator were excluded for the analysis of that respective indicator. The sample selection process is detailed in S1 Fig.

### Maternal age and marital status

Maternal age at the time of the recorded birth was categorized as <18, 18–19, and 20–24 years [19,20,22]. Marital status was a binary indicator identifying mothers married at the time of conception or delivery or anytime in between. It was self-reported by the mother in all states and DC, except New York, which applied an inferential approach for all or some of the births recorded between 2014 and 2019. In New York, a birth was automatically recorded by the data provider as marital unless a paternity acknowledgement had been received or the father’s name was missing [33–38]. Because of state statutory restrictions preventing the release of marital information, all births recorded in California between 2017 and 2019 have missing marital status [38]. Since these statutory restrictions are responsible for 98.9% of observations with missing marital status (S1 Fig), most births with missing marital information were recorded between 2017 and 2019 (S1 Table). There was no difference in maternal age group proportions between the study sample and births with missing marital status; a higher proportion of births with missing marital information had mothers who were Hispanic or foreign-born and mothers who had received Special Supplemental Nutrition Program for Women, Infants, and Children (WIC) benefits, and a lower proportion had missing paternal information and mothers who were white or black (S1 Table).

### Reproductive, maternal, and infant health indicators

The definitions of the indicators used as dependent variables are presented in Table 1. Reproductive health indicators included prior pregnancy termination, repeat birth, any maternal smoking during pregnancy, and late or no initiation of prenatal care. Maternal health indicators comprised diagnosis for sexually transmitted infection (STI) during pregnancy, gestational hypertension, eclampsia, and maternal morbidity. Infant health indicators were preterm birth, SGA, infant morbidity, and no breastfeeding at discharge or time of birth certificate completion.

**Table 1:** Description of the reproductive, maternal, and infant health indicators.

Indicator	Description
*Reproductive health*	
Prior pregnancy termination	At least 1 previous ectopic pregnancy, spontaneous loss, and/or induced abortion
Repeat birth	Parity ≥ 1 before the recorded birth
Maternal smoking	Any smoking during the pregnancy of the recorded birth (first, second, and/or third trimester)
Late/no prenatal care initiation	Prenatal care was initiated in the second or third trimester of the pregnancy of the recorded birth or was never initiated
*Maternal health*	
Sexually transmitted infection	Presence of or treatment received for any of the following 5 sexually transmitted infections during the pregnancy of the recorded birth: gonorrhea, syphilis, chlamydia, hepatitis B, and/or hepatitis C
Gestational hypertension	Presence of gestational hypertension (pregnancy-induced hypertension or preeclampsia) during the pregnancy of the recorded birth
Eclampsia	Presence of eclampsia during the pregnancy of the recorded birth
Maternal morbidity	Presence of any of the following 5 conditions during or after delivery: maternal transfusion, third or fourth degree perineal laceration, ruptured uterus, unplanned hysterectomy, and/or admission to intensive care unit
*Infant health*	
Preterm	Recorded birth occurred before 37 completed weeks of gestation
Small for gestational age	Birth weight < 10th percentile for gestational age using the sex-specific INTERGROWTH-21st (International Fetal and Newborn Growth Consortium for the 21st Century) birth weight charts for infants born at 24–32 or 33–42 completed weeks of gestation [41]
Infant morbidity	Presence of any of the following 6 conditions after birth: assisted ventilation required immediately after birth, assisted ventilation required for >6 hours, admission to neonatal intensive care unit, administration of surfactant replacement therapy, administration of antibiotics for suspected neonatal sepsis, and/or seizure or serious neurological dysfunction
Infant not breastfed at discharge	No breastfeeding at time of hospital discharge or time of birth certificate completion, whichever comes first (includes intent to breastfeed but with no initiation by the time of discharge or birth certificate completion)

### Covariates

Covariates were identified a priori based on availability in the data and the literature on child marriage [5,12,13,15–18] and on reproductive outcomes among teens [19–22,39]. The analysis of all indicators accounted for maternal race/ethnicity recorded using the race and Hispanic origin categories listed in the 2003 US Standard Certificate of Live Birth, maternal nativity status, paternal age, receipt of WIC benefits, whether the delivery was primarily paid for by Medicaid, and birth year. Receipt of WIC and Medicaid benefits represented proxy indicators of low socioeconomic status [42]. Birth year was included to account for observation exclusions due to data unavailability in certain years and for temporal variations in the proportion of marital births, teen birth rate, and the indicators [24–26,43–47]. The analysis of certain indicators also accounted for maternal smoking during pregnancy, parity, any diabetes, preexisting hypertension, prenatal care adequacy [48], and infant sex. All covariates were categorical, and a “not available” category was created for those with missing data.

### Statistical analyses

The characteristics of marital and nonmarital births within maternal age groups were compared using absolute standardized differences [49]. Multivariable logistic regression models were conducted to examine the joint associations of marital status and maternal age group with reproductive, maternal, and infant health indicators, while controlling for covariates. Each model included an interaction term between marital status and maternal age group and a set of covariates that differed based on their relevance to the indicator. Following best practice for reporting results for an interaction between 2 independent variables, 3 sets of adjusted odds ratios (AORs) and their 95 confidence intervals (95% CIs) were estimated for each indicator [50]: (1) joint AORs for marital status and maternal age group with births to unmarried mothers aged 20–24 years as the reference group, (2) AORs by maternal age group within each marital status, and (3) AORs by marital status within each maternal age group. The description of the results primarily focuses on the second set of AORs, to examine whether the age gradient in the indicators differed between births to married and unmarried mothers, and on the third set of AORs, to examine whether the association between marriage and the indicators differed between births to mothers aged <18, 18–19, and 20–24 years. The 3 sets of unadjusted odds ratios and 95% CIs are presented for each indicator in S1 File.

Two exploratory analyses were conducted. First, since the propensity to stop smoking during pregnancy may vary by marital status and maternal age, AORs for maternal smoking during the third trimester of pregnancy were estimated and compared to those for any maternal smoking during pregnancy (S2 File). Then, births to mothers aged <18 years were stratified by maternal age < 16 and 16–17 years to explore differences in the age gradients and the association with marriage between the 2 age groups (S3 File). Studies suggest mothers aged <16 and 16–17 years have different risks of certain reproductive outcomes [19,20,22], but given the small number of births to married mothers aged <16 years (*n* = 363), all births to mothers below age 18 were combined for the primary analysis.

Several sensitivity analyses were also conducted to test the robustness of the results. First, the impact of including births recorded in 2014 and 2015, when the 2003 birth certificate was not nationally implemented, was assessed by running separate models for births recorded in 2014–2015 and 2016–2019 (S4 File). Second, maternal education was added as a covariate to examine its influence on the association between marriage and the indicators (S5 File), given the important relationship documented between child marriage and education in LMICs [11,13]. The primary analysis did not include maternal education because the levels measured in birth certificates appeared to be mainly a function of age among young mothers and were insufficient to determine if mothers aged <18 years had achieved age-appropriate education levels. Third, the impact of maternal pre-pregnancy body mass index (BMI) was also assessed for the indicators gestational hypertension, eclampsia, maternal morbidity, preterm birth, SGA, and infant morbidity (S6 File). Fourth, paternal age was replaced by paternal education to test if accounting for this marker of socioeconomic status changed the results (S7 File); paternal age and education were not included together because of multicollinearity. Finally, paternal age was replaced by parental age gap (paternal age minus maternal age) (S8 File), a covariate included in some studies on child marriage and reproductive health in LMICs [12,16]; paternal age was selected over parental age gap to differentiate adolescent and adult fathers in the primary analysis.

All analyses were performed in SAS V9.4.

## Results

Marital births represented 3.6% of the 314,098 births to mothers aged <18 years, compared to 13.2% of the 873,111 births to mothers aged 18–19 years and 34.1% of the 4,482,615 births to mothers aged 20–24 years (Table 2). For all maternal age groups, a higher proportion of marital births had white, Asian, or foreign-born mothers, and a lower proportion had black mothers and unavailable paternal age. In the <18 years age group, a higher proportion of marital births had Hispanic mothers, older fathers, and mothers with high school graduation/General Education Development (GED). In the older age groups, a lower proportion of marital births received WIC and Medicaid benefits, and a higher proportion had mothers with education levels above high school graduation/GED.

**Table 2:** Characteristics of births to US mothers aged ≤24 years by maternal age group and marital status, 2014–2019 (*n* = 5,669,824)*.

Characteristics	Maternal age <18 years (*n* = 314,098)			Maternal age 18–20 years (*n* = 873,111)			Maternal age 20–24 years (*n* = 4,482,615)		
	Nonmarital births (*n* = 302,933), *n* (%)	Marital births (*n* = 11,165), *n* (%)	*|d|*	Nonmarital births (*n* = 758,259), *n* (%)	Marital births (*n* = 114,852), *n* (%)	*|d|*	Nonmarital births (*n* = 2,953,099), *n* (%)	Marital births (*n* = 1,529,516), *n* (%)	*|d|*
Maternal race/ethnicity									
White	93,814 (31.0)	5,125 (45.9)	0.31	293,981 (38.8)	61,068 (53.2)	0.29	1,170,451 (39.6)	927,772 (60.7)	0.43
Black	72,239 (23.9)	330 (3.0)	0.64	175,942 (23.2)	5,536 (4.8)	0.55	791,144 (26.8)	101,249 (6.6)	0.56
American Indian & Alaska Native	5,165 (1.7)	81 (0.7)	0.09	11,735 (1.6)	973 (0.9)	0.06	41,306 (1.4)	11,172 (0.7)	0.07
Asian	1,996 (0.7)	326 (2.9)	0.17	5,325 (0.7)	2,828 (2.5)	0.14	31,176 (1.1)	59,372 (3.9)	0.18
Native Hawaiian & Pacific Islander	621 (0.2)	25 (0.2)	0.00	1,998 (0.3)	335 (0.3)	0.01	8,631 (0.3)	4,780 (0.3)	0.00
Multi-race	11,293 (3.7)	222 (2.0)	0.10	27,506 (3.6)	2,729 (2.4)	0.07	95,273 (3.2)	32,483 (2.1)	0.07
Hispanic^†^	116,177 (38.4)	5,002 (44.8)	0.13	238,167 (31.4)	40,799 (35.5)	0.09	800,998 (27.1)	383,838 (25.1)	0.05
Not available	1,628 (0.5)	54 (0.5)	0.01	3,605 (0.5)	584 (0.5)	0.00	14,120 (0.5)	8,850 (0.6)	0.01
Maternal nativity status									
US-born	266,758 (88.1)	8,329 (74.6)	0.35	675,431 (89.1)	91,703 (79.8)	0.26	2,603,069 (88.2)	1,227,597 (80.3)	0.22
Foreign-born	35,366 (11.7)	2,814 (25.2)	0.35	81,119 (10.7)	22,902 (19.9)	0.26	343,626 (11.6)	298,919 (19.5)	0.22
Not available	809 (0.3)	22 (0.2)	0.01	1,709 (0.2)	247 (0.2)	0.00	6,404 (0.2)	3,000 (0.2)	0.00
Paternal age									
<18 years	49,955 (16.5)	979 (8.8)	0.23	20,603 (2.7)	874 (0.8)	0.15	4,112 (0.1)	404 (0.03)	0.04
18–19 years	66,161 (21.8)	3,732 (33.4)	0.26	144,513 (19.1)	19,916 (17.3)	0.04	68,767 (2.3)	14,119 (0.9)	0.11
20–24 years	40,201 (13.3)	4,768 (42.7)	0.69	281,406 (37.1)	69,882 (60.9)	0.49	1,056,063 (35.8)	650,308 (42.5)	0.14
≥25 years	5,938 (2.0)	1,496 (13.4)	0.44	72,971 (9.6)	22,250 (19.4)	0.28	984,282 (33.3)	830,580 (54.3)	0.43
Not available	140,678 (46.4)	190 (1.7)	1.23	238,766 (31.5)	1,930 (1.7)	0.87	839,875 (28.4)	34,105 (2.2)	0.78
WIC received									
No	73,357 (24.2)	2,837 (25.4)	0.03	195,543 (25.8)	39,125 (34.1)	0.18	988,134 (33.5)	834,766 (54.6)	0.44
Yes	224,859 (74.2)	8,140 (72.9)	0.03	551,449 (72.7)	73,998 (64.4)	0.18	1,918,799 (65.0)	670,632 (43.9)	0.43
Not available	4,717 (1.6)	188 (1.7)	0.01	11,267 (1.5)	1,729 (1.5)	0.00	46,166 (1.6)	24,118 (1.6)	0.00
Medicaid as main payor									
No	62,896 (20.8)	2,531 (22.7)	0.05	167,702 (22.1)	38,408 (33.4)	0.25	800,349 (27.1)	819,056 (53.6)	0.56
Yes	237,660 (78.5)	8,526 (76.4)	0.05	585,000 (77.2)	75,395 (65.7)	0.26	2,132,257 (72.2)	696,998 (45.6)	0.56
Not available	2,377 (0.8)	108 (1.0)	0.02	5,557 (0.7)	1,049 (0.9)	0.02	20,493 (0.7)	13,462 (0.9)	0.02
Birth year									
2014	65,104 (21.5)	2,858 (25.6)	0.10	152,917 (20.2)	25,229 (22.0)	0.04	563,196 (19.1)	296,494 (19.4)	0.01
2015	60,345 (19.9)	2,621 (23.5)	0.09	143,839 (19.0)	22,868 (19.9)	0.02	552,902 (18.7)	286,899 (18.8)	0.00
2016	54,880 (18.1)	2,082 (18.7)	0.01	134,261 (17.7)	20,742 (18.1)	0.01	529,012 (17.9)	274,610 (18.0)	0.00
2017	43,979 (14.5)	1,535 (13.8)	0.02	114,326 (15.1)	17,275 (15.0)	0.00	451,548 (15.3)	235,442 (15.4)	0.00
2018	40,515 (13.4)	1,153 (10.3)	0.09	107,652 (14.2)	15,132 (13.2)	0.03	432,269 (14.6)	222,965 (14.6)	0.00
2019	38,110 (12.6)	916 (8.2)	0.14	105,264 (13.9)	13,606 (11.9)	0.06	424,172 (14.4)	213,106 (13.9)	0.01
Infant sex									
Male	154,997 (51.2)	5,783 (51.8)	0.01	388,692 (51.3)	59,386 (51.7)	0.01	1,509,741 (51.1)	783,589 (51.2)	0.00
Female	147,936 (48.8)	5,382 (48.2)	0.01	369,567 (48.7)	55,466 (48.3)	0.01	1,443,358 (48.9)	745,927 (48.8)	0.00
Any maternal smoking									
No	282,930 (93.4)	10,398 (93.1)	0.01	672,127 (88.6)	105,308 (91.7)	0.10	2,529,949 (85.7)	1,418,743 (92.8)	0.23
Yes	16,604 (5.5)	685 (6.1)	0.03	77,620 (10.2)	8,489 (7.4)	0.10	388,129 (13.1)	97,293 (6.4)	0.23
Not available	3,399 (1.1)	82 (0.7)	0.04	8,512 (1.1)	1,055 (0.9)	0.02	35,021 (1.2)	13,480 (0.9)	0.03
Parity									
0	281,031 (92.8)	9,515 (85.2)	0.24	613,969 (81.0)	86,118 (75.0)	0.14	1,556,263 (52.7)	750,123 (49.0)	0.07
1	19,684 (6.5)	1,436 (12.9)	0.22	122,793 (16.2)	24,212 (21.1)	0.13	919,486 (31.1)	533,126 (34.9)	0.08
≥2	1,353 (0.5)	181 (1.6)	0.12	18,968 (2.5)	4,131 (3.6)	0.06	466,915 (15.8)	240,595 (15.7)	0.00
Not available	865 (0.3)	33 (0.3)	0.00	2,529 (0.3)	391 (0.3)	0.00	10,435 (0.4)	5,672 (0.4)	0.00
Any diabetes^‡^									
No	296,965 (98.0)	10,914 (97.8)	0.02	737,704 (97.3)	111,333 (96.9)	0.02	2,838,920 (96.1)	1,461,767 (95.6)	0.03
Yes	5,559 (1.8)	239 (2.1)	0.02	19,611 (2.6)	3,391 (3.0)	0.02	110,487 (3.7)	65,890 (4.3)	0.03
Not available	409 (0.1)	12 (0.1)	0.01	944 (0.1)	128 (0.1)	0.00	3,692 (0.1)	1,859 (0.1)	0.00
Preexisting hypertension									
No	300,995 (99.4)	11,101 (99.4)	0.01	751,568 (99.1)	113,944 (99.2)	0.01	2,915,359 (98.7)	1,512,078 (98.9)	0.01
Yes	1,529 (0.5)	52 (0.5)	0.00	5,747 (0.8)	780 (0.7)	0.01	34,048 (1.2)	15,579 (1.0)	0.01
Not available	409 (0.1)	12 (0.1)	0.01	944 (0.1)	128 (0.1)	0.00	3,692 (0.1)	1,859 (0.1)	0.00
Prenatal care adequacy^§^									
No care	11,514 (3.8)	327 (2.9)	0.05	20,788 (2.7)	2,214 (1.9)	0.05	77,163 (2.6)	20,026 (1.3)	0.09
Intensive	16,335 (5.4)	639 (5.7)	0.01	44,867 (5.9)	7,154 (6.2)	0.01	191,457 (6.5)	102,877 (6.7)	0.01
Adequate	78,965 (26.1)	3,448 (30.9)	0.11	239,583 (31.6)	40,091 (34.9)	0.07	1,016,217 (34.4)	612,490 (40.0)	0.12
Intermediate	130,630 (43.1)	4,653 (41.7)	0.03	318,425 (42.0)	47,210 (41.1)	0.02	1,185,948 (40.2)	605,285 (39.6)	0.01
Inadequate	54,593 (18.0)	1,732 (15.5)	0.07	108,461 (14.3)	14,656 (12.8)	0.05	380,174 (12.9)	144,965 (9.5)	0.11
Not available	10,896 (3.6)	366 (3.3)	0.02	26,135 (3.5)	3,527 (3.1)	0.02	102,140 (3.5)	43,873 (2.9)	0.03
Maternal education^#^									
≤8th grade	26,193 (8.7)	937 (8.4)	0.01	18,795 (2.5)	4,753 (4.1)	0.09	66,733 (2.3)	56,285 (3.7)	0.08
9th–12th grade, no diploma	236,483 (78.1)	7,624 (68.3)	0.22	250,559 (33.0)	30,053 (26.2)	0.15	490,424 (16.6)	154,152 (10.1)	0.19
High school graduate/GED	34,820 (11.5)	2,206 (19.8)	0.23	381,418 (50.3)	58,983 (51.4)	0.02	1,358,123 (46.0)	561,290 (36.7)	0.19
Above high school/GED	1,837 (0.6)	210 (1.9)	0.11	100,759 (13.3)	19,963 (17.4)	0.11	1,012,684 (34.3)	746,150 (48.8)	0.30
Not available	3,600 (1.2)	188 (1.7)	0.04	6,728 (0.9)	1,100 (1.0)	0.01	25,135 (0.9)	11,639 (0.8)	0.01
Maternal pre-pregnancy BMI									
Underweight (<18.5 kg/m^2^)	24,251 (8.0)	972 (8.7)	0.03	51,594 (6.8)	7,701 (6.7)	0.00	146,326 (5.0)	68,670 (4.5)	0.02
Normal weight (18.5–24.9 kg/m^2^)	164,910 (54.4)	5,842 (52.3)	0.04	362,830 (47.9)	54,694 (47.6)	0.00	1,206,031 (40.8)	660,967 (43.2)	0.05
Overweight (25.0–29.9 kg/m^2^)	63,957 (21.1)	2,450 (21.9)	0.02	173,435 (22.9)	26,803 (23.3)	0.01	715,788 (24.2)	376,842 (24.6)	0.01
Obesity (≥30 kg/m^2^)	39,352 (13.0)	1,531 (13.7)	0.02	148,311 (19.6)	22,681 (19.8)	0.00	797,564 (27.0)	386,754 (25.3)	0.04
Not available	10,463 (3.5)	370 (3.3)	0.01	22,089 (2.9)	2,973 (2.6)	0.02	87,390 (3.0)	36,283 (2.4)	0.04
Paternal education^#^									
≤8th grade	7,588 (2.5)	857 (7.7)	0.24	17,036 (2.3)	6,498 (5.7)	0.18	69,052 (2.3)	73,419 (4.8)	0.13
9th–12th grade, no diploma	84,753 (28.0)	3,726 (33.4)	0.12	144,036 (19.0)	21,996 (19.2)	0.00	369,011 (12.5)	162,072 (10.6)	0.06
High school graduate/GED	54,647 (18.0)	4,846 (43.4)	0.57	263,902 (34.8)	58,318 (50.8)	0.33	1,032,284 (35.0)	597,441 (39.1)	0.08
Above high school/GED	8,092 (2.7)	1,328 (11.9)	0.36	74,411 (9.8)	24,481 (21.3)	0.32	558,894 (18.9)	643,832 (42.1)	0.52
Not available	147,853 (48.8)	408 (3.7)	1.20	258,874 (34.1)	3,559 (3.1)	0.87	923,858 (31.3)	52,752 (3.5)	0.79
Parental age gap									
Mother older than father	7,604 (2.5)	121 (1.1)	0.11	41,950 (5.5)	3,291 (2.9)	0.13	282,660 (9.6)	131,345 (8.6)	0.03
Father 0–2 years older	96,146 (31.7)	4,015 (36.0)	0.09	267,370 (35.3)	52,051 (45.3)	0.21	892,245 (30.2)	672,005 (43.9)	0.29
Father 3–4 years older	37,746 (12.5)	3,142 (28.1)	0.40	102,760 (13.6)	26,126 (22.8)	0.24	373,569 (12.7)	296,319 (19.4)	0.18
Father 5–9 years older	17,257 (5.7)	2,738 (24.5)	0.54	79,366 (10.5)	23,023 (20.1)	0.27	385,142 (13.0)	285,413 (18.7)	0.15
Father ≥10 years older	3,502 (1.2)	959 (8.6)	0.35	28,047 (3.7)	8,431 (7.3)	0.16	179,608 (6.1)	110,329 (7.2)	0.05
Not available	140,678 (46.4)	190 (1.7)	1.23	238,766 (31.5)	1,930 (1.7)	0.87	839,875 (28.4)	34,105 (2.2)	0.78

Column percent may not add to 100% because of rounding. *|d|* is the absolute standardized difference between births to married and unmarried mothers within the maternal age group.

*Includes all births to US mothers aged ≤24 years with data recorded using the 2003 US Standard Certificate of Live Birth and with available marital status.

^†^Includes all mothers who self-identified as Hispanic with or without another race/ethnicity.

^‡^Preexisting or gestational diabetes.

^§^Derived using the Revised Graduated Prenatal Care Utilization Index (Revised-GINDEX) [48].

^#^When the level of maternal educational attainment was not compatible with maternal age (i.e., educational level too high for maternal age), the education level was categorized as “Not available” as per the consistency checks applied by the Division of Vital Statistics in the 2018 and 2019 Natality Public Use Files [37,38]. The consistency checks were applied to paternal education for all years.

BMI, body mass index; GED, General Education Development; WIC, Special Supplemental Nutrition Program for Women, Infants, and Children.

**Fig 1:**
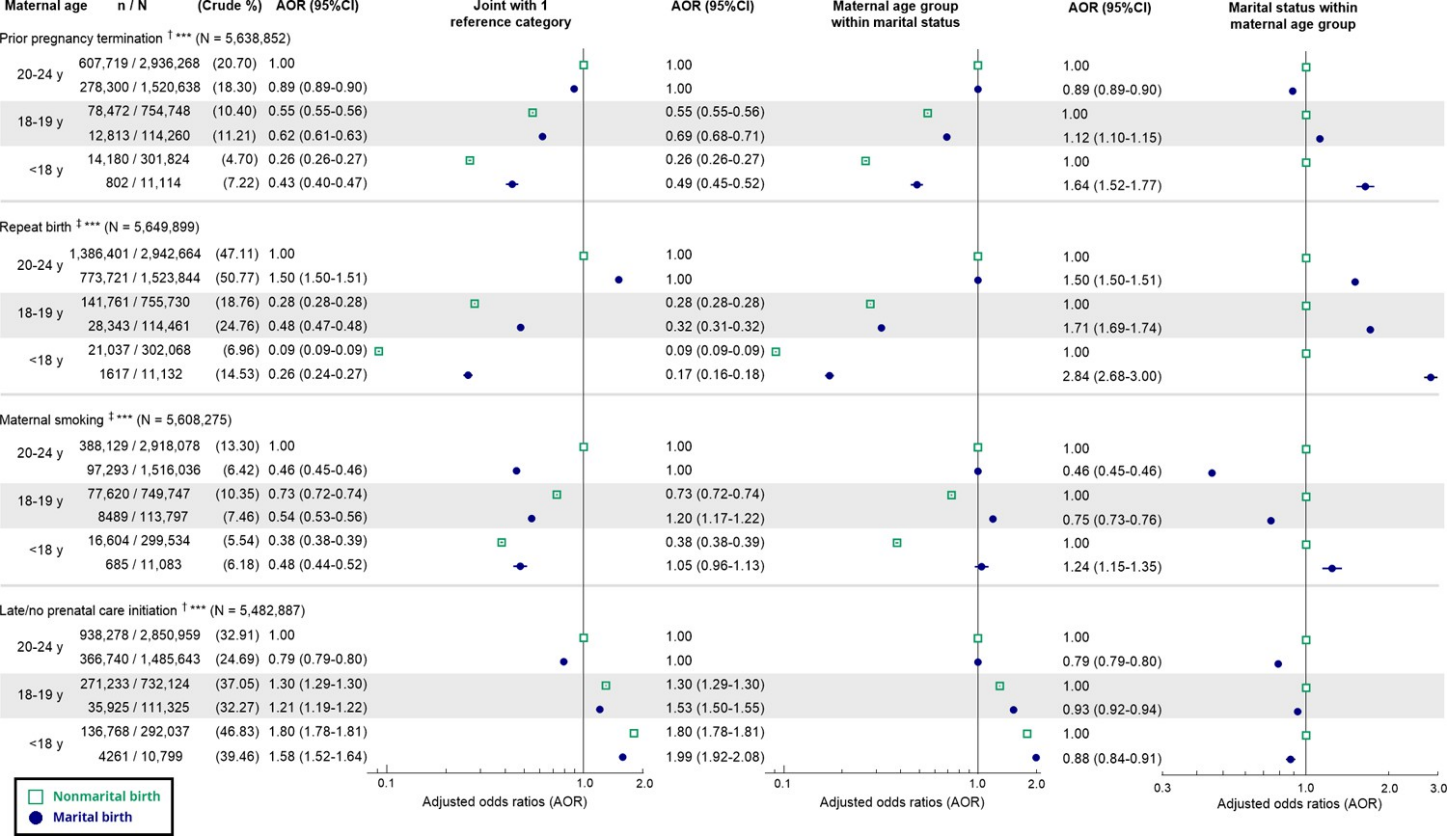
Adjusted odds ratios of reproductive health indicators associated with the interaction between marital status and maternal age group. AOR plotted on the logarithmic scale. ^†^Adjusted for maternal race/ethnicity, maternal US-born status, parity, paternal age, WIC benefits received, Medicaid as main payor of the delivery, and birth year. ^‡^Adjusted for maternal race/ethnicity, maternal US-born status, paternal age, WIC benefits received, Medicaid as main payor of the delivery, and birth year. ***p < 0.001 for interaction term between marital status and maternal age group. AOR, adjusted odds ratio; WIC, Special Supplemental Nutrition Program for Women, Infants, and Children.

A statistically significant interaction was present between marital status and maternal age group for all reproductive health indicators (Fig 1). Compared to births to unmarried mothers aged 20–24 years, all births had lower AORs of prior pregnancy termination and maternal smoking, while only births to younger married and unmarried mothers had lower AORs of repeat birth and higher AORs of late or no prenatal care initiation. Within each marital status, the AOR of prior pregnancy termination and repeat birth declined with younger maternal age, but the age gradient was steeper among nonmarital births. The AOR of maternal smoking declined with younger maternal age among nonmarital births, but among marital births, the adjusted odds of maternal smoking was higher in the 18–19 years age group (AOR 1.20, 95% CI 1.17–1.22, *p <* 0.001) and similar in the <18 years age group (AOR 1.05, 95% CI 0.96–1.13, *p* = 0.29) relative to the oldest group. Within each marital status, the AOR of late or no prenatal care initiation increased with younger maternal age, with a steeper age gradient among marital births. Among births to mothers aged <18 years, marriage was associated with higher adjusted odds of prior pregnancy termination (AOR 1.64, 95% CI 1.52–1.77, *p <* 0.001), repeat birth (AOR 2.84, 95% CI 2.68–3.00, *p <* 0.001), and maternal smoking (AOR 1.24, 95% CI 1.15–1.35, *p <* 0.001); within the older groups, the AORs for these indicators were closer to the null or reversed. Within each maternal age group, marriage was associated with lower adjusted odds of late or no prenatal care initiation, but the association was weaker among births to mothers aged <18 (AOR 0.88, 95% CI 0.84–0.91, *p <* 0.001) and 18–19 years (AOR 0.93, 95% CI 0.92–0.94, *p <* 0.001) than among births to mothers aged 20–24 years (AOR 0.79, 95% CI 0.79–0.80, *p <* 0.001).

**Fig 2:**
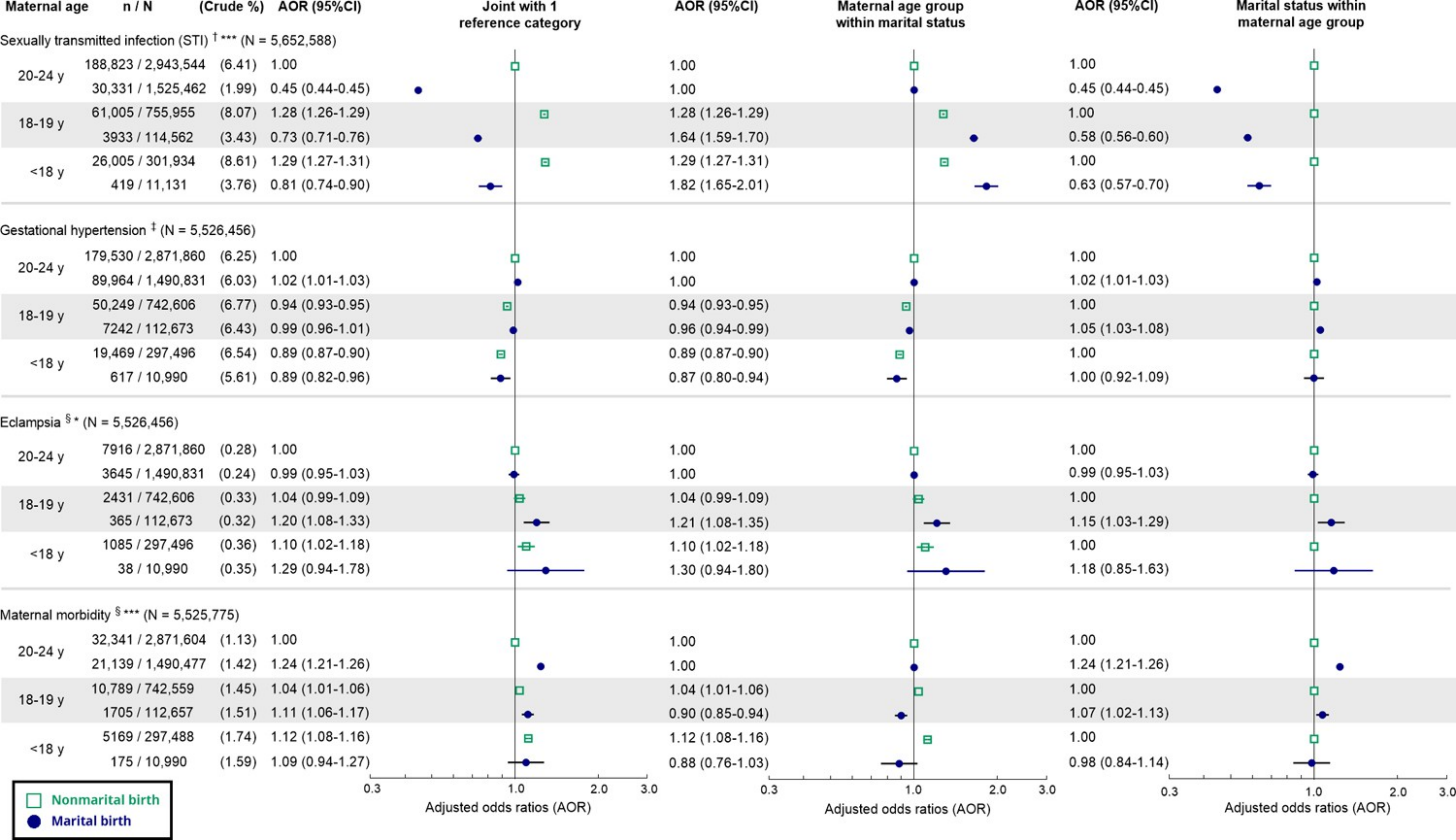
Adjusted odds ratios of maternal health indicators associated with the interaction between marital status and maternal age group. AOR plotted on the logarithmic scale. ^†^Adjusted for maternal race/ethnicity, maternal US-born status, parity, paternal age, WIC benefits received, Medicaid as main payor of the delivery, and birth year. ^‡^Adjusted for maternal race/ethnicity, maternal US-born status, parity, maternal smoking, prenatal care adequacy, any diabetes (preexisting or gestational), paternal age, WIC benefits received, Medicaid as main payor of the delivery, and birth year. ^§^Adjusted for maternal race/ethnicity, maternal US-born status, parity, maternal smoking, prenatal care adequacy, any diabetes (preexisting or gestational), preexisting hypertension, paternal age, WIC benefits received, Medicaid as main payor of the delivery, and birth year. *p < 0.05, ***p < 0.001 for interaction term between marital status and maternal age group. AOR, adjusted odds ratio; WIC, Special Supplemental Nutrition Program for Women, Infants, and Children.

For the maternal health indicators, there was a significant interaction between marital status and maternal age group for STI, eclampsia, and maternal morbidity but not gestational hypertension (Fig 2). Compared to births to unmarried mothers aged 20–24 years, all marital births had lower AORs of STI. Within each marital status, births to mothers aged <18 and 18–19 years had higher adjusted odds of STI than those to mothers aged 20–24 years, but the AORs were higher among marital births. Irrespective of marital status, the AOR of gestational hypertension declined slightly with younger maternal age. Eclampsia was very uncommon (<0.4%), and within each marital status, the AOR tended to increase with younger maternal age, but only the AORs for births to married mothers aged 18–19 years (AOR 1.21, 95% CI 1.08–1.35, *p <* 0.001) and to unmarried mothers aged <18 years (AOR 1.10, 95% CI 1.02–1.18, *p* = 0.01) were statistically significant. The AOR of maternal morbidity increased with younger maternal age among nonmarital births, whereas for marital births, the AOR was lower among births to mothers aged 18–19 years (AOR 0.90, 95% CI 0.85–0.94, *p <* 0.001) and statistically non-significant among births to mothers aged <18 years (AOR 0.88, 95% CI 0.76–1.03, *p* = 0.11). Within each maternal age group, marriage was associated with lower adjusted odds of STI, but the association was weaker among births to mothers aged <18 (AOR 0.63, 95% CI 0.57–0.70, *p <* 0.001) and 18–19 years (AOR 0.58, 95% CI 0.56–0.60, *p <* 0.001) than among births to mothers aged 20–24 years (AOR 0.45, 95% CI 0.44–0.45, *p <* 0.001). Among births to mothers aged <18 years, no association was found between marriage and gestational hypertension, eclampsia, or maternal morbidity; marriage was associated with elevated AORs of all 3 indicators among births to mothers aged 18–19 years and with higher AORs of gestational hypertension and maternal morbidity among births to mothers aged 20–24 years.

**Fig 3:**
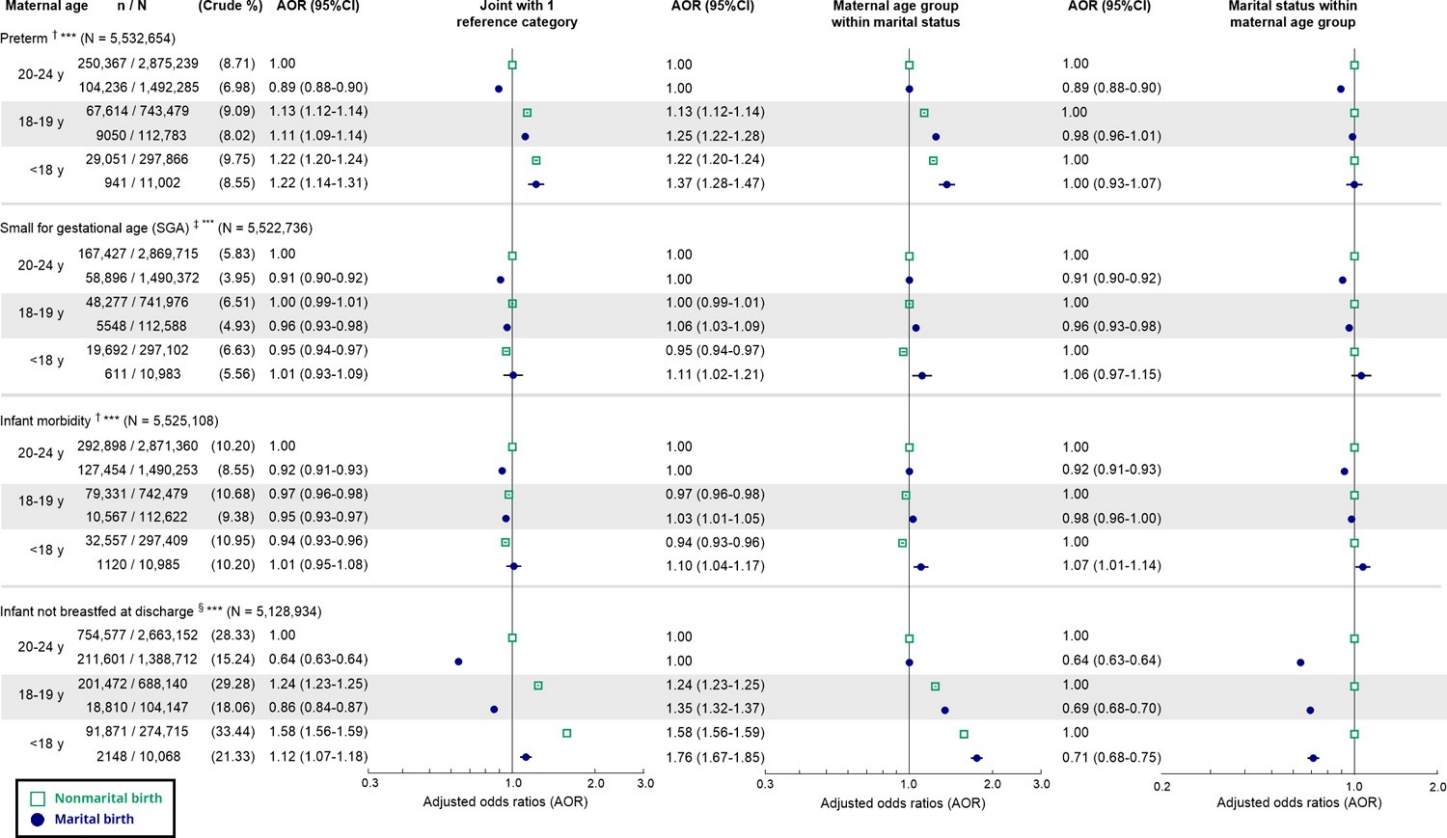
Adjusted odds ratios of infant health indicators associated with the interaction between marital status and maternal age group. AOR plotted on the logarithmic scale. ^†^Adjusted for infant sex, maternal race/ethnicity, maternal US-born status, parity, maternal smoking, prenatal care adequacy, any diabetes (preexisting or gestational), preexisting hypertension, paternal age, WIC benefits received, Medicaid as main payor of the delivery, and birth year. ^‡^Adjusted for maternal race/ethnicity, maternal US-born status, parity, maternal smoking, prenatal care adequacy, any diabetes (preexisting or gestational), preexisting hypertension, paternal age, WIC benefits received, Medicaid as main payor of the delivery, and birth year. ^§^Adjusted for maternal race/ethnicity, maternal US-born status, parity, maternal smoking, prenatal care adequacy, paternal age, WIC benefits received, Medicaid as main payor of the delivery, and birth year. ***p < 0.001 for interaction term between marital status and maternal age. AOR, adjusted odds ratio; WIC, Special Supplemental Nutrition Program for Women, Infants, and Children.

The interaction between marital status and maternal age was statistically significant for all infant health indicators (Fig 3). Compared to births to unmarried mothers aged 20–24 years, births to younger married and unmarried mothers had higher AORs of being preterm; greater AORs of no breastfeeding at discharge were only present among births to unmarried mothers aged 18–19 years and those to unmarried and married mothers aged <18 years. Within each marital status, the adjusted odds of preterm birth and no breastfeeding at discharge increased with younger maternal age, with steeper age gradients among marital births. The AORs of SGA and infant morbidity tended to decline with younger maternal age among nonmarital births but tended to increase with younger maternal age among marital births. Marriage was not associated with preterm birth or SGA among births to mothers aged <18 years, but it was associated with marginally or significantly lower adjusted odds of both indicators within the older age groups. There was a small positive association between marriage and infant morbidity among births to mothers aged <18 years (AOR 1.07, 95% CI 1.01–1.14, *p* = 0.03), while the association was reversed within the older groups. Within each maternal age group, marriage was associated with lower adjusted odds of no breastfeeding at discharge, but the association was weaker among births to mothers aged <18 (AOR 0.71, 95% CI 0.68–0.75, *p <* 0.001) and 18–19 years (AOR 0.69, 95% CI 0.68–0.70, *p <* 0.001) than among births to mothers aged 20–24 years (AOR 0.64, 95% CI 0.63–0.64, *p <* 0.001).

### Results from the exploratory and sensitivity analyses

Maternal smoking during the third trimester was less prevalent than any maternal smoking during pregnancy for marital and nonmarital births in all maternal age groups (S2 File). The patterns in the age gradients within marital status and in the association with marriage within maternal age groups were very similar for the 2 smoking indicators, suggesting that smoking cessation by the third trimester was generally proportional across marital status and maternal age groups.

When differentiating births to mothers aged <16 and 16–17 years (S3 File), the age gradients obtained within marital status in the primary analysis generally continued in these age groups, meaning AORs that decreased (increased) with younger maternal age tended to be lower (higher) among the <16 years age group than the 16–17 years age group for many indicators. The association of marriage with greater adjusted odds of prior pregnancy termination (AOR 2.87, 95% CI 1.75–4.70, *p <* 0.001), repeat birth (AOR 7.08, 95% CI 4.93–10.17, *p <* 0.001), and maternal smoking (AOR 2.32, 95% CI 1.40–3.86, *p* = 0.001) was stronger among births to mothers <16 years than among those to mothers aged 16–17 years. Marriage was associated with lower adjusted odds of STI and no breastfeeding at discharge in all age groups and with lower adjusted odds of late or no prenatal care initiation in all except the <16 years age group (AOR 1.06, 95% CI 0.85–1.31, *p* = 0.61). Marriage was not associated with gestational hypertension, eclampsia, maternal morbidity, preterm birth, or SGA among births to mothers aged <16 and 16–17 years, but it was associated with marginally higher adjusted odds of infant morbidity among births to mothers aged 16–17 years (AOR 1.08, 95% CI 1.01–1.15, *p* = 0.02).

The sensitivity analyses led to some movements in the AORs of the indicators, but the overall result patterns and their interpretations were similar to the ones obtained in the primary analyses, except for a few notable differences. Among births to mothers aged 20–24 years, marriage was associated with higher AOR of eclampsia for births recorded in 2014–2015 (AOR 1.13, 95% CI 1.05–1.21, *p* = 0.002), but it was associated with lower AOR for births recorded in 2016–2019 (AOR 0.93, 95% CI 0.88–0.98, *p* = 0.006), resulting in a non-significant association when combining all years (AOR 0.99, 95% CI 0.95–1.03, *p* = 0.67) (S4 File). The inclusion of maternal education (S5 File) and replacement of paternal age with paternal education (S7 File) both resulted in lower AORs of maternal smoking among births to married mothers aged <18 and 18–19 years compared to births to married mothers aged 20–24 years; however, the AORs associated with marriage remained similar within each age group because the age gradient among births to unmarried mothers became steeper. The inclusion of maternal education also resulted in the disappearance of the small age gradient in the AORs of SGA and infant morbidity among marital births (S5 File). Including maternal pre-pregnancy BMI resulted in statistically non-significant AORs for gestational hypertension for all marital status and maternal age groups, and in the disappearance of the small age gradient in the AOR of SGA among marital births (S6 File). Finally, replacing paternal age with parental age gap resulted in lower AOR of maternal smoking among births to married mothers aged <18 years compared to births to married mothers aged 20–24 years (AOR 0.88, 95% CI 0.81–0.95, *p* = 0.001), and the association between marriage and greater odds of maternal smoking appeared slightly weaker among births to mothers aged <18 years (AOR 1.14, 95% CI 1.05–1.24, *p* = 0.002) (S8 File).

## Discussion

Using recent population-based birth records, we found that marriage among US mothers below age 18 years was associated with greater odds of prior pregnancy termination, repeat birth, maternal smoking, and infant morbidity, after covariate control. These findings diverge from the weaker or reverse associations observed among mothers aged 18–19 and 20–24 years for the same indicators. For mothers aged 20–24 years, marriage was associated with lower odds of several indicators, including late or no prenatal care initiation, STI, preterm birth, SGA, and no breastfeeding at discharge. Yet, for mothers aged <18 and 18–19 years, these beneficial associations were weaker or absent.

Research in LMICs suggests that women who marry before age 18 are more likely to experience pregnancy termination [13,15–18] and higher fertility [12,13,15,18] than those who marry at or after age 18. By comparing married and unmarried mothers below age 18, our study identifies an association between girl child marriage and higher odds of pregnancy termination and repeat early childbearing in the US. Different mechanisms could underlie these relationships. Married adolescents below age 18 may face unbalanced power dynamics and may have limited ability to negotiate contraceptive use and sexual intercourse frequency, resulting in high early fertility [13,51]. Alternatively, married adolescents below age 18 and their husbands may perceive family growth as a desirable life trajectory, leading to intended early pregnancies [51]. Life circumstances, such as childhood socioeconomic disadvantages or limited prospects for education and work, could also contribute simultaneously to marriage and fertility among female adolescents below age 18 [39,52,53]. Lastly, a US study suggests that the relationship between early childbearing and early marriage is bidirectional [39]. It found that married teens have greater odds of a first birth before age 20, and teens with a first birth have greater odds of getting married before age 20 [39]. Because of a lack of information on the timing of marriage relative to prior pregnancy events, it was not possible in our study to disentangle these 2 mechanisms among mothers below age 18 and those aged 18–19 and 20–24 years. While more research is required to better understand the underlying pathways, our results signal that marriage before age 18 has a distinct relationship with fertility compared to marriage among older adolescents in the US.

The strong relationship between child marriage and early childbearing is considered an important public health concern because of heightened risk of poor maternal and infant outcomes among young mothers [13,18,20,22]. Our results suggest that relative to their older counterparts, married and unmarried mothers below age 18 had greater adjusted odds of late or no prenatal care initiation, STI, preterm birth, and not breastfeeding. Depending on their marital status, mothers below age 18 also had marginally higher adjusted odds of SGA and maternal and infant morbidity compared to mothers aged 20–24 years. Our findings also revealed that the relationships between maternal age and several indicators vary substantially by marital status, suggesting that research on reproductive health among adolescent mothers should consider the influence of marital status in country contexts where both marital and extramarital childbearing occur.

The comparison of married and unmarried mothers below age 18 indicates child marriage is not consistently associated with greater vulnerability to adverse reproductive, maternal, and infant health indicators. Marriage among mothers below age 18 was associated with lower adjusted odds of late or no prenatal care initiation, STI, and not breastfeeding, albeit these beneficial associations were weaker than those estimated among mothers aged 20–24 years. These findings may be indicative of more stable and supportive monogamous relationships among married than unmarried mothers below age 18 [28,29]. Consistent with prior analyses from high-income countries [27–30], we found lower adjusted odds of preterm birth, SGA, and maternal smoking among married mothers aged 20–24 years compared to their unmarried counterparts, but our findings also suggest that these favorable associations with marriage do not apply to mothers below age 18. The association between marriage and higher odds of smoking among mothers below age 18 may suggest that those who marry at a young age have a greater tendency towards riskier behaviors [28,52], although the beneficial association of marriage with early prenatal care initiation and breastfeeding also indicates an inclination toward certain health-promoting behaviors. Overall, the patterns depicted in this study indicate great heterogeneity in the association between marital status and reproductive, maternal, and infant health indicators across adolescent maternal age groups. They highlight the importance of not generalizing findings from older adolescent mothers or the general population to those below age 18, and of differentiating younger and older adolescents in research, when possible.

Despite lower marriage rates relative to several decades ago, marriage remains a highly valued social practice that influences the legal, social, and economic status of women and families in the US [54,55]. Marriage has long been perceived as a societal ideal associated with better socioeconomic, health, and child outcomes [54]. However, research indicates that marriage before age 16 increases the probability of experiencing poverty in later life among US women [53]. Other studies have also found that US women who marry before age 18 or 19 have greater risk of subsequently developing poor mental health [56] and chronic conditions [57]. The notion that girl child marriage represents a unique phenomenon that must be examined and understood separately from marriage among women aged 18 or older is supported by our findings, those of previous studies [53,56,57], and the exceptional context of marriage before age 18 in the US [23,58]. Given that marrying at a young age is associated with marital instability [5,53,56] and that high early fertility can limit maternal education and labor opportunities [59], married mothers below age 18 and their children may be particularly vulnerable to future adverse health outcomes [56,57] and socioeconomic hardships [53,54]. Public knowledge regarding child marriage is very limited in the US [58], and little research has been conducted on the topic domestically [5,7], but as most states permit marriage below age 18 [8], it is critical to understand the extent of the health and social implications of child marriage in the country. Our study contributes to addressing this important knowledge gap, but additional work using longitudinal designs is required to examine other health and social indicators in relation to child marriage and the underlying pathways. More research is also needed to develop an understanding of the contemporary drivers of child marriage in the US and other high-income countries [5,7].

### Limitations

The analyses relied on cross-sectional data with no information on the timing of marriage formation relative to prior fertility events or the pregnancy of the recorded birth. Our analytic approach is consistent with indications that marriage is a predictor of first childbirth among US adolescents, but prior pregnancy events may have also occurred before marriage [39]. Information on pregnancy intention and the cause of any prior pregnancy termination was also unavailable. Little is known about the drivers of contemporary child marriages in the US [5,7], and research is needed to determine to what extent intended or unintended pregnancies and births contribute to child marriage formation. The data available precluded the identification of mothers aged 18–19 and 20–24 years who married before turning 18. Not identifying these mothers likely had minimal impact on the association between marital status and the indicators within the 2 age groups, since child marriage is uncommon in the US [5]. Common-law unions could also not be identified in the data. Studies indicate single mothers have greater risk of adverse birth outcomes than those in common-law unions in the general population [29,30]. Future research using comprehensive data on parental relationship and marital status is needed to determine whether these findings extend to mothers below age 18. Despite accounting for several covariates in the analyses, unobserved differences by marital status and maternal age may contribute to residual confounding [29,30,39,54]. Religiosity [39] and geographic information, including state of residence and rurality, were not accounted for due to unavailable data. Information on maternal education was also insufficient to create a covariate for age-appropriate education level for mothers below age 18. Recall bias and social desirability bias are possible for the information reported by mothers, including marital status. The inferential approach used for marital status in the state of New York also likely contributed to misclassification error. Data quality studies suggest that several of the dependent variables and covariates used in the analyses have good accuracy and/or agreement when compared to data from medical records, hospital discharge records, or the Pregnancy Risk Assessment Monitoring System, but those identifying medical conditions, including STI, gestational hypertension, eclampsia, and maternal morbidity, are underreported [42]. The impact of underreporting on the results is unclear, since it is unknown whether underreporting varies by marital status or adolescent maternal age [42]. Finally, the study sample was not fully representative of all 50 states and DC from 2014 to 2019, because observations were excluded from states where the 2003 birth certificate had not been fully implemented in 2014–2015 and from California in 2017–2019, where marital status information was missing. Nevertheless, this study represents a novel population-based examination of the association between marriage before age 18 and several reproductive, maternal, and infant health indicators in the US. In contrast to previous research in LMICs that only compared women married before and after turning 18 [12,14–18], our analyses compared married and unmarried mothers less than 18 years and contrasted associations across age groups, providing a broader picture of the interplay between adolescent maternal age and marital status in contemporary US.

### Conclusion

This study adds to the limited understanding of the reproductive implications of girl child marriage in the US. It suggests that marriage below age 18 is associated with both adverse and favorable reproductive, maternal, and infant health indicators among US mothers. Our findings highlight the importance of distinguishing the determinants and consequences of child marriage from those associated with marriage at older ages. A better understanding of the health and social consequences and the driving forces of child marriage in high-income settings is critical to help shape context-relevant responses.

## Supporting information

S1 Checklist STROBE checklist for cross-sectional studies. (DOC)

S1 Fig Sample selection process. (PDF)

S1 File Unadjusted and adjusted odds ratios of reproductive, maternal, and infant health indicators associated with the interaction between marital status and maternal age group: Primary analysis. (PDF)

S2 File Adjusted odds ratios of any maternal smoking during pregnancy and maternal smoking in third trimester associated with the interaction between marital status and maternal age group: Exploratory analysis. (PDF)

S3 File Adjusted odds ratios of reproductive, maternal, and infant health indicators associated with the interaction between marital status and maternal age group: Exploratory analysis with maternal age groups <16 and 16–17 years. (PDF)

S4 File Adjusted odds ratios of reproductive, maternal, and infant health indicators associated with the interaction between marital status and maternal age group: Sensitivity analysis for births recorded in 2014–2015 and in 2016–2019. (PDF)

S5 File Adjusted odds ratios of reproductive, maternal, and infant health indicators associated with the interaction between marital status and maternal age group: Sensitivity analysis with maternal education. (PDF)

S6 File Adjusted odds ratios of reproductive, maternal, and infant health indicators associated with the interaction between marital status and maternal age group: Sensitivity analysis with pre-pregnancy body mass index (BMI). (PDF)

S7 File Adjusted odds ratios of reproductive, maternal, and infant health indicators associated with the interaction between marital status and maternal age group: Sensitivity analysis with paternal education. (PDF)

S8 File Adjusted odds ratios of reproductive, maternal, and infant health indicators associated with the interaction between marital status and maternal age group: Sensitivity analysis with parental age gap. (PDF)

S1 Table Characteristics of births to US mothers aged ≤24 years by marital status availability. (PDF)

## Abbreviations

AOR: adjusted odds ratio
BMI: body mass index
DC: District of Columbia
GED: General Education Development
LMICs: low- and middle-income countries
SGA: small for gestational age
STI: sexually transmitted infection
WIC: Special Supplemental Nutrition Program for Women, Infants, and Children
