# Supplementary material for: Reproductive health among married and unmarried mothers aged less than 18, 18–19, and 20–24 years in the United States, 2014–2019: A population-based cross-sectional study
Source: PLoS Med. 2022 Mar 10;19(3):e1003929. doi: 10.1371/journal.pmed.1003929 (PMC8912259; doi:10.1371/journal.pmed.1003929)
Supplement: S1 Fig — (PDF) [file pmed.1003929.s002.pdf]

S1 Figure. Study sample selection process

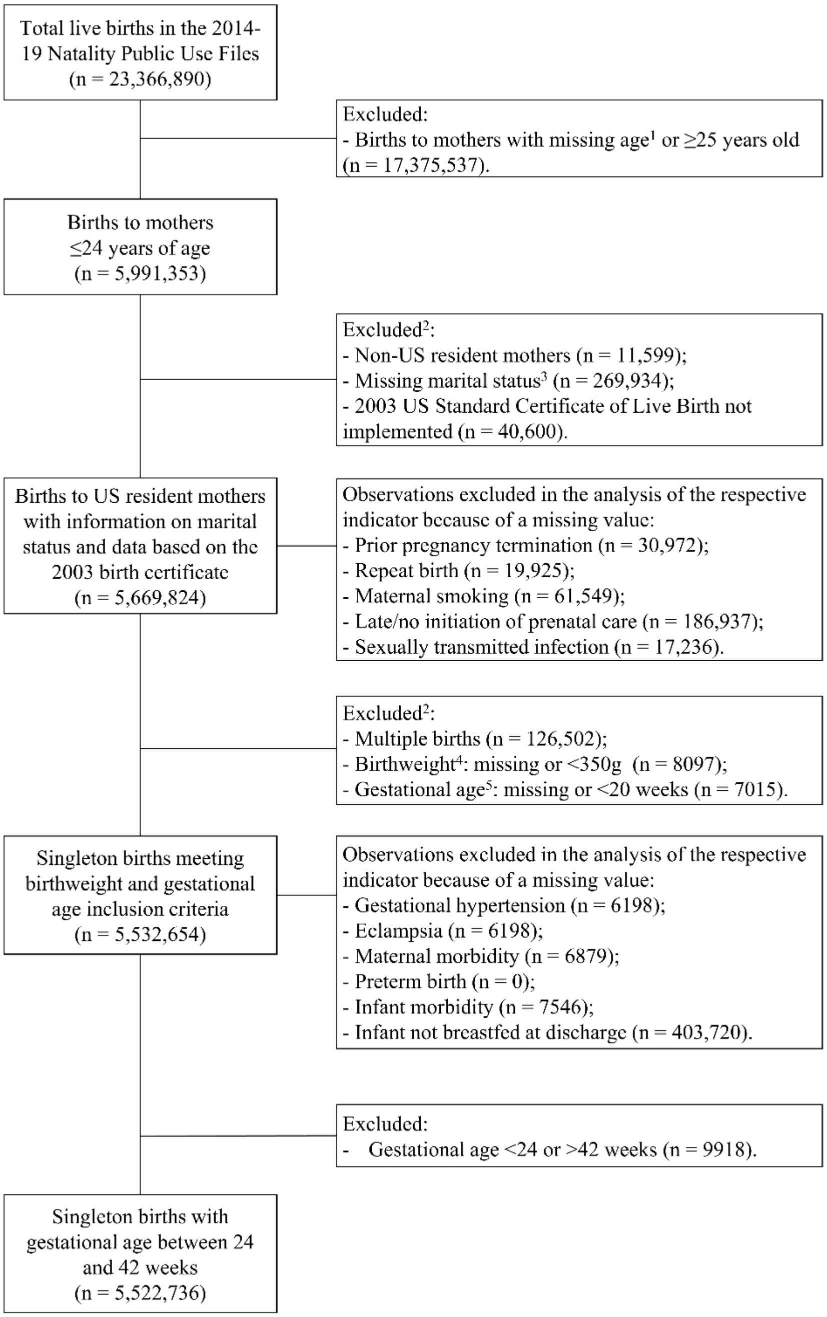

[1] 2834 observations had missing maternal age, representing 0.01% of all birth records from January 2014 to December 2019.
[2] Observations can meet more than one exclusion criteria.
[3] Of these excluded observations, 98.9% were birth records from the state of California (n = 266,887), where the release of marital status is not permitted since January 2017.
[4] Of these excluded observations, 60.2% had a missing birthweight value (n = 4875).
[5] Of these excluded observations, 69.0% had a missing gestational age value (n = 4841).
