# Supplementary material for: Reproductive health among married and unmarried mothers aged less than 18, 18–19, and 20–24 years in the United States, 2014–2019: A population-based cross-sectional study
Source: PLoS Med. 2022 Mar 10;19(3):e1003929. doi: 10.1371/journal.pmed.1003929 (PMC8912259; doi:10.1371/journal.pmed.1003929)
Supplement: S2 File — (PDF) [file pmed.1003929.s004.pdf]

S2 File. Adjusted odds ratios of any maternal smoking during pregnancy and maternal smoking in 3rd trimester associated with the interaction between marital status and maternal age group, exploratory analysis

	Any maternal smoking during pregnancy as dependent variable primary analysis				Maternal smoking during 3rd trimester as dependent variable sensitivity analysis			
	Crude %	Adjusted odds ratios (95%CI) †			Crude %	Adjusted odds ratios (95%CI) †		
Marital status & maternal age group		Joint with 1 reference category	By maternal age group within marital status	By marital status within maternal age group		Joint with 1 reference category	By maternal age group within marital status	By marital status within maternal age group
		***				***		
Unmarried 20-24y	13.30	1.00	1.00	1.00	10.25	1.00	1.00	1.00
Married 20-24y	6.42	0.46 (0.45-0.46)	1.00	0.46 (0.45-0.46)	5.04	0.49 (0.49-0.50)	1.00	0.49 (0.49-0.50)
Unmarried 18-19y	10.35	0.73 (0.72-0.74)	0.73 (0.72-0.74)	1.00	7.52	0.69 (0.69-0.70)	0.69 (0.69-0.70)	1.00
Married 18-19y	7.46	0.54 (0.53-0.56)	1.20 (1.17-1.22)	0.75 (0.73-0.76)	5.59	0.55 (0.54-0.57)	1.12 (1.09-1.15)	0.79 (0.77-0.82)
Unmarried <18y	5.54	0.38 (0.38-0.39)	0.38 (0.38-0.39)	1.00	3.88	0.36 (0.35-0.37)	0.36 (0.35-0.37)	1.00
Married <18y	6.18	0.48 (0.44-0.52)	1.05 (0.96-1.13)	1.24 (1.15-1.35)	4.75	0.50 (0.45-0.54)	1.01 (0.92-1.10)	1.38 (1.26-1.51)

† Adjusted for maternal race/ethnicity, US-born status, paternal age, WIC received, Medicaid as main payor of the delivery, and birth year.
* p < 0.05, ** p <0.01, *** p <0.001 for interaction term between marital status and maternal age group.
