# Supplementary material for: Reproductive health among married and unmarried mothers aged less than 18, 18–19, and 20–24 years in the United States, 2014–2019: A population-based cross-sectional study
Source: PLoS Med. 2022 Mar 10;19(3):e1003929. doi: 10.1371/journal.pmed.1003929 (PMC8912259; doi:10.1371/journal.pmed.1003929)
Supplement: S3 File — (PDF) [file pmed.1003929.s005.pdf]

S3 File. Adjusted odds ratios of reproductive, maternal, and infant health indicators associated with the interaction between marital status and maternal age group, exploratory analysis with maternal age <16 and 16-17 years

**Reproductive health indicator**	**N**	**n**	**Crude %**	**Adjusted odds ratios (95%CI)**		
Marital status & maternal age group				Joint with 1 reference category	By maternal age group within marital status	By marital status within maternal age group
Prior pregnancy termination † ***						
Unmarried 20-24y	2,936,268	607,719	20.70	1.00	1.00	1.00
Married 20-24y	1,520,638	278,300	18.30	0.89 (0.89-0.90)	1.00	0.89 (0.89-0.90)
Unmarried 18-19y	754,748	78,472	10.40	0.55 (0.55-0.56)	0.55 (0.55-0.56)	1.00
Married 18-19y	114,260	12,813	11.21	0.62 (0.61-0.63)	0.69 (0.68-0.71)	1.12 (1.10-1.15)
Unmarried 16-17y	254,059	13,261	5.22	0.29 (0.29-0.30)	0.29 (0.29-0.30)	1.00
Married 16-17y	10,754	785	7.30	0.44 (0.41-0.47)	0.49 (0.46-0.53)	1.49 (1.38-1.61)
Unmarried <16y	47,765	919	1.92	0.11 (0.10-0.11)	0.11 (0.10-0.11)	1.00
Married <16y	360	17	4.72	0.30 (0.19-0.50)	0.34 (0.21-0.56)	2.87 (1.75-4.70)
Repeat birth ‡ ***						
Unmarried 20-24y	2,942,664	1,386,401	47.11	1.00	1.00	1.00
Married 20-24y	1,523,844	773,721	50.77	1.50 (1.50-1.51)	1.00	1.50 (1.50-1.51)
Unmarried 18-19y	755,730	141,761	18.76	0.28 (0.28-0.28)	0.28 (0.28-0.28)	1.00
Married 18-19y	114,461	28,343	24.76	0.48 (0.47-0.48)	0.32 (0.31-0.32)	1.71 (1.69-1.74)
Unmarried 16-17y	254,280	20,054	7.89	0.11 (0.10-0.11)	0.11 (0.10-0.11)	1.00
Married 16-17y	10,770	1583	14.70	0.26 (0.25-0.28)	0.17 (0.17-0.18)	2.47 (2.33-2.61)
Unmarried <16y	47,788	983	2.06	0.02 (0.02-0.02)	0.02 (0.02-0.02)	1.00
Married <16y	362	34	9.39	0.16 (0.11-0.23)	0.11 (0.08-0.16)	7.08 (4.93-10.17)
Maternal smoking ‡ ***						
Unmarried 20-24y	2,918,078	388,129	13.30	1.00	1.00	1.00
Married 20-24y	1,516,036	97,293	6.42	0.46 (0.45-0.46)	1.00	0.46 (0.45-0.46)
Unmarried 18-19y	749,747	77,620	10.35	0.73 (0.72-0.74)	0.73 (0.72-0.74)	1.00
Married 18-19y	113,797	8489	7.46	0.54 (0.53-0.56)	1.20 (1.17-1.22)	0.75 (0.73-0.76)
Unmarried 16-17y	252,173	15,155	6.01	0.42 (0.41-0.43)	0.42 (0.41-0.43)	1.00
Married 16-17y	10,722	668	6.23	0.48 (0.44-0.52)	1.04 (0.97-1.14)	1.14 (1.05-1.24)
Unmarried <16y	47,361	1449	3.06	0.20 (0.19-0.21)	0.20 (0.19-0.21)	1.00
Married <16y	361	17	4.71	0.46 (0.28-0.77)	1.01 (0.61-1.68)	2.32 (1.40-3.86)
Late/no prenatal care initiation † ***						
Unmarried 20-24y	2,850,959	938,278	32.91	1.00	1.00	1.00
Married 20-24y	1,485,643	366,740	24.69	0.79 (0.79-0.80)	1.00	0.79 (0.79-0.80)
Unmarried 18-19y	732,124	271,233	37.05	1.30 (1.29-1.30)	1.30 (1.29-1.30)	1.00
Married 18-19y	111,325	35,925	32.27	1.21 (1.19-1.22)	1.53 (1.50-1.55)	0.93 (0.92-0.94)
Unmarried 16-17y	245,942	110,270	44.84	1.69 (1.67-1.70)	1.69 (1.67-1.70)	1.00
Married 16-17y	10,452	4070	38.94	1.55 (1.49-1.61)	1.96 (1.88-2.04)	0.92 (0.88-0.96)
Unmarried <16y	46,095	26,498	57.49	2.58 (2.53-2.63)	2.58 (2.53-2.63)	1.00
Married <16y	347	191	55.04	2.73 (2.20-3.38)	3.45 (2.79-4.28)	1.06 (0.85-1.31)

† Adjusted for maternal race/ethnicity, US-born status, parity, paternal age, WIC received, Medicaid as main payor of the delivery, and birth year.
‡ Adjusted for maternal race/ethnicity, US-born status, paternal age, WIC received, Medicaid as main payor of the delivery, and birth year.
* $p < 0.05$, ** $p < 0.01$, *** $p < 0.001$ for interaction term between marital status and maternal age group.

Maternal health indicator	N	n	Crude %	Adjusted odds ratios (95%CI)		
Marital status & maternal age group				Joint with 1 reference category	By maternal age group within marital status	By marital status within maternal age group
Sexually transmitted infection (STI) † ***						
Unmarried 20-24y	2,943,544	188,823	6.41	1.00	1.00	1.00
Married 20-24y	1,525,462	30,331	1.99	0.45 (0.44-0.45)	1.00	0.45 (0.44-0.45)
Unmarried 18-19y	755,955	61,005	8.07	1.28 (1.26-1.29)	1.28 (1.26-1.29)	1.00
Married 18-19y	114,562	3933	3.43	0.73 (0.71-0.76)	1.64 (1.59-1.70)	0.58 (0.56-0.60)
Unmarried 16-17y	254,220	21,903	8.62	1.31 (1.29-1.33)	1.31 (1.29-1.33)	1.00
Married 16-17y	10,768	410	3.81	0.82 (0.75-0.91)	1.84 (1.67-2.04)	0.63 (0.57-0.70)
Unmarried <16y	47,714	4102	8.60	1.16 (1.12-1.20)	1.16 (1.12-1.20)	1.00
Married <16y	363	9	2.48	0.54 (0.28-1.05)	1.21 (0.63-2.35)	0.47 (0.24-0.91)
Gestational hypertension ‡						
Unmarried 20-24y	2,871,860	179,530	6.25	1.00	1.00	1.00
Married 20-24y	1,490,831	89,964	6.03	1.02 (1.01-1.03)	1.00	1.02 (1.01-1.03)
Unmarried 18-19y	742,606	50,249	6.77	0.94 (0.93-0.95)	0.94 (0.93-0.95)	1.00
Married 18-19y	112,673	7242	6.43	0.99 (0.96-1.01)	0.96 (0.94-0.99)	1.05 (1.03-1.08)
Unmarried 16-17y	250,367	16,291	6.51	0.88 (0.87-0.90)	0.88 (0.87-0.90)	1.00
Married 16-17y	10,637	600	5.64	0.89 (0.82-0.97)	0.87 (0.80-0.94)	1.00 (0.92-1.09)
Unmarried <16y	47,129	3178	6.74	0.91 (0.88-0.95)	0.91 (0.88-0.95)	1.00
Married <16y	353	17	4.82	0.83 (0.51-1.36)	0.81 (0.50-1.33)	0.91 (0.56-1.49)
Eclampsia §						
Unmarried 20-24y	2,871,860	7916	0.28	1.00	1.00	1.00
Married 20-24y	1,490,831	3645	0.24	0.99 (0.95-1.03)	1.00	0.99 (0.95-1.03)
Unmarried 18-19y	742,606	2431	0.33	1.04 (0.99-1.09)	1.04 (0.99-1.09)	1.00
Married 18-19y	112,673	365	0.32	1.20 (1.08-1.33)	1.21 (1.08-1.35)	1.15 (1.03-1.29)
Unmarried 16-17y	250,367	894	0.36	1.08 (1.01-1.17)	1.08 (1.01-1.17)	1.00
Married 16-17y	10,637	37	0.35	1.30 (0.94-1.80)	1.31 (0.95-1.82)	1.20 (0.86-1.67)
Unmarried <16y	47,129	191	0.41	1.17 (1.00-1.35)	1.17 (1.00-1.35)	1.00
Married <16y	353	1	0.28	1.05 (0.15-7.47)	1.06 (0.15-7.54)	0.90 (0.13-6.44)
Maternal morbidity § ***						
Unmarried 20-24y	2,871,604	32,341	1.13	1.00	1.00	1.00
Married 20-24y	1,490,477	21,139	1.42	1.24 (1.21-1.26)	1.00	1.24 (1.21-1.26)
Unmarried 18-19y	742,559	10,789	1.45	1.04 (1.01-1.06)	1.04 (1.01-1.06)	1.00
Married 18-19y	112,657	1705	1.51	1.11 (1.06-1.17)	0.90 (0.85-0.94)	1.07 (1.02-1.13)
Unmarried 16-17y	250,345	4212	1.68	1.09 (1.06-1.13)	1.09 (1.06-1.13)	1.00
Married 16-17y	10,636	169	1.59	1.09 (0.94-1.28)	0.88 (0.76-1.03)	1.00 (0.86-1.17)
Unmarried <16y	47,143	957	2.03	1.24 (1.16-1.33)	1.24 (1.16-1.33)	1.00
Married <16y	354	6	1.69	1.09 (0.49-2.44)	0.88 (0.39-1.97)	0.88 (0.39-1.97)

† Adjusted for maternal race/ethnicity, US-born status, parity, paternal age, WIC received, Medicaid as main payor of the delivery, and birth year.

‡ Adjusted for maternal race/ethnicity, US-born status, parity, maternal smoking, prenatal care adequacy, any diabetes (pre-existing or gestational), paternal age, WIC received, Medicaid as main payor of the delivery, and birth year.

§ Adjusted for maternal race/ethnicity, US-born status, parity, maternal smoking, prenatal care adequacy, any diabetes (pre-existing or gestational), pre-existing hypertension, paternal age, WIC received, Medicaid as main payor of the delivery, and birth year.

* $p < 0.05$, ** $p < 0.01$, *** $p < 0.001$ for interaction term between marital status and maternal age group.

Infant health indicator	N	n	Crude %	Adjusted odds ratios (95%CI)		
Marital status & maternal age group				Joint with 1 reference category	By maternal age group within marital status	By marital status within maternal age group
Preterm † ***						
Unmarried 20-24y	2,875,239	250,367	8.71	1.00	1.00	1.00
Married 20-24y	1,492,285	104,236	6.98	0.89 (0.88-0.90)	1.00	0.89 (0.88-0.90)
Unmarried 18-19y	743,479	67,614	9.09	1.13 (1.12-1.14)	1.13 (1.12-1.14)	1.00
Married 18-19y	112,783	9050	8.02	1.11 (1.09-1.14)	1.25 (1.22-1.28)	0.98 (0.96-1.01)
Unmarried 16-17y	250,654	23720	9.46	1.19 (1.17-1.21)	1.19 (1.17-1.21)	1.00
Married 16-17y	10,648	904	8.49	1.21 (1.13-1.30)	1.36 (1.27-1.46)	1.02 (0.95-1.09)
Unmarried <16y	47,212	5331	11.29	1.41 (1.37-1.46)	1.41 (1.37-1.46)	1.00
Married <16y	354	37	10.45	1.52 (1.07-2.15)	1.70 (1.20-2.41)	1.07 (0.76-1.52)
Small for gestational age (SGA) ‡ ***						
Unmarried 20-24y	2,869,715	167,427	5.83	1.00	1.00	1.00
Married 20-24y	1,490,372	58,896	3.95	0.91 (0.90-0.92)	1.00	0.91 (0.90-0.92)
Unmarried 18-19y	741,976	48,277	6.51	1.00 (0.99-1.01)	1.00 (0.99-1.01)	1.00
Married 18-19y	112,588	5548	4.93	0.96 (0.93-0.98)	1.06 (1.03-1.09)	0.96 (0.93-0.98)
Unmarried 16-17y	250,047	16,634	6.65	0.97 (0.95-0.99)	0.97 (0.95-0.99)	1.00
Married 16-17y	10,630	590	5.55	1.01 (0.93-1.10)	1.11 (1.02-1.21)	1.04 (0.96-1.13)
Unmarried <16y	47,055	3058	6.50	0.86 (0.83-0.90)	0.86 (0.83-0.90)	1.00
Married <16y	353	21	5.95	0.99 (0.64-1.55)	1.10 (0.70-1.71)	1.15 (0.74-1.80)
Infant morbidity † ***						
Unmarried 20-24y	2,871,360	292,898	10.20	1.00	1.00	1.00
Married 20-24y	1,490,253	127,454	8.55	0.92 (0.91-0.93)	1.00	0.92 (0.91-0.93)
Unmarried 18-19y	742,479	79,331	10.68	0.97 (0.96-0.98)	0.97 (0.96-0.98)	1.00
Married 18-19y	112,622	10,567	9.38	0.95 (0.93-0.97)	1.03 (1.01-1.05)	0.98 (0.96-1.00)
Unmarried 16-17y	250,281	26,926	10.76	0.94 (0.92-0.95)	0.94 (0.92-0.95)	1.00
Married 16-17y	10,632	1080	10.16	1.01 (0.95-1.08)	1.10 (1.03-1.17)	1.08 (1.01-1.15)
Unmarried <16y	47,128	5631	11.95	0.99 (0.96-1.02)	0.99 (0.96-1.02)	1.00
Married <16y	353	40	11.33	1.09 (0.78-1.52)	1.19 (0.85-1.65)	1.10 (0.79-1.53)
Infant not breastfed at discharge § ***						
Unmarried 20-24y	2,663,152	754,577	28.33	1.00	1.00	1.00
Married 20-24y	1,388,712	211,601	15.24	0.64 (0.63-0.64)	1.00	0.64 (0.63-0.64)
Unmarried 18-19y	688,140	201,472	29.28	1.24 (1.23-1.25)	1.24 (1.23-1.25)	1.00
Married 18-19y	104,147	18,810	18.06	0.86 (0.84-0.87)	1.35 (1.32-1.37)	0.69 (0.68-0.70)
Unmarried 16-17y	231,052	74,623	32.30	1.51 (1.50-1.53)	1.51 (1.50-1.53)	1.00
Married 16-17y	9756	2075	21.27	1.11 (1.06-1.17)	1.75 (1.66-1.84)	0.74 (0.70-0.77)
Unmarried <16y	43,663	17,248	39.50	1.99 (1.95-2.03)	1.99 (1.95-2.03)	1.00
Married <16y	312	73	23.40	1.34 (1.02-1.75)	2.10 (1.61-2.75)	0.67 (0.51-0.88)

† Adjusted for infant sex, maternal race/ethnicity, US-born status, parity, maternal smoking, prenatal care adequacy, any diabetes (pre-existing or gestational), pre-existing hypertension, paternal age, WIC received, Medicaid as main payor of the delivery, and birth year.
‡ Adjusted for maternal race/ethnicity, US-born status, parity, maternal smoking, prenatal care adequacy, any diabetes (pre-existing or gestational), pre-existing hypertension, paternal age, WIC received, Medicaid as main payor of the delivery, and birth year.
§ Adjusted for maternal race/ethnicity, US-born status, parity, maternal smoking, prenatal care adequacy, paternal age, WIC received, Medicaid as main payor of the delivery, and birth year.
* $p < 0.05$, ** $p < 0.01$, *** $p < 0.001$ for interaction term between marital status and maternal age group.
