# Supplementary material for: Reproductive health among married and unmarried mothers aged less than 18, 18–19, and 20–24 years in the United States, 2014–2019: A population-based cross-sectional study
Source: PLoS Med. 2022 Mar 10;19(3):e1003929. doi: 10.1371/journal.pmed.1003929 (PMC8912259; doi:10.1371/journal.pmed.1003929)
Supplement: S4 File — (PDF) [file pmed.1003929.s006.pdf]

S4 File. Adjusted odds ratios of reproductive, maternal, and infant health indicators associated with the interaction between marital status and maternal age group, sensitivity analysis for births recorded in 2014-2015 and in 2016-2019

**Reproductive health indicator**	**Adjusted odds ratios (95%CI), primary analysis**			**Adjusted odds ratios (95%CI) §, sensitivity analysis for births recorded in 2014-2015**			**Adjusted odds ratios (95%CI) §, sensitivity analysis for births recorded in 2016-2019**		
Marital status & maternal age group	Joint with 1 reference category	By maternal age group within marital status	By marital status within maternal age group	Joint with 1 reference category	By maternal age group within marital status	By marital status within maternal age group	Joint with 1 reference category	By maternal age group within marital status	By marital status within maternal age group
Prior pregnancy termination †	***			***			***		
Unmarried 20-24y	1.00	1.00	1.00	1.00	1.00	1.00	1.00	1.00	1.00
Married 20-24y	0.89 (0.89-0.90)	1.00	0.89 (0.89-0.90)	0.89 (0.88-0.90)	1.00	0.89 (0.88-0.90)	0.90 (0.89-0.90)	1.00	0.90 (0.89-0.90)
Unmarried 18-19y	0.55 (0.55-0.56)	0.55 (0.55-0.56)	1.00	0.55 (0.54-0.55)	0.55 (0.54-0.55)	1.00	0.55 (0.55-0.56)	0.55 (0.55-0.56)	1.00
Married 18-19y	0.62 (0.61-0.63)	0.69 (0.68-0.71)	1.12 (1.10-1.15)	0.60 (0.58-0.62)	0.68 (0.66-0.70)	1.10 (1.06-1.13)	0.63 (0.62-0.65)	0.70 (0.69-0.72)	1.14 (1.11-1.17)
Unmarried <18y	0.26 (0.26-0.27)	0.26 (0.26-0.27)	1.00	0.27 (0.26-0.27)	0.27 (0.26-0.27)	1.00	0.26 (0.26-0.27)	0.26 (0.26-0.27)	1.00
Married <18y	0.43 (0.40-0.47)	0.49 (0.45-0.52)	1.64 (1.52-1.77)	0.41 (0.37-0.46)	0.46 (0.42-0.52)	1.56 (1.39-1.73)	0.45 (0.41-0.50)	0.51 (0.46-0.56)	1.72 (1.56-1.91)
Repeat birth ‡	***			***			***		
Unmarried 20-24y	1.00	1.00	1.00	1.00	1.00	1.00	1.00	1.00	1.00
Married 20-24y	1.50 (1.50-1.51)	1.00	1.50 (1.50-1.51)	1.55 (1.54-1.56)	1.00	1.55 (1.54-1.56)	1.48 (1.47-1.48)	1.00	1.48 (1.47-1.48)
Unmarried 18-19y	0.28 (0.28-0.28)	0.28 (0.28-0.28)	1.00	0.28 (0.28-0.29)	0.28 (0.28-0.29)	1.00	0.28 (0.28-0.28)	0.28 (0.28-0.28)	1.00
Married 18-19y	0.48 (0.47-0.48)	0.32 (0.31-0.32)	1.71 (1.69-1.74)	0.49 (0.48-0.50)	0.32 (0.31-0.32)	1.74 (1.70-1.78)	0.47 (0.46-0.48)	0.32 (0.31-0.32)	1.69 (1.66-1.72)
Unmarried <18y	0.09 (0.09-0.09)	0.09 (0.09-0.09)	1.00	0.09 (0.09-0.09)	0.09 (0.09-0.09)	1.00	0.09 (0.09-0.09)	0.09 (0.09-0.09)	1.00
Married <18y	0.26 (0.24-0.27)	0.17 (0.16-0.18)	2.84 (2.68-3.00)	0.26 (0.24-0.28)	0.17 (0.16-0.18)	2.81 (2.60-3.04)	0.26 (0.24-0.28)	0.18 (0.16-0.19)	2.85 (2.64-3.09)
Maternal smoking ‡	***			***			***		
Unmarried 20-24y	1.00	1.00	1.00	1.00	1.00	1.00	1.00	1.00	1.00
Married 20-24y	0.46 (0.45-0.46)	1.00	0.46 (0.45-0.46)	0.48 (0.47-0.48)	1.00	0.48 (0.47-0.48)	0.44 (0.44-0.45)	1.00	0.44 (0.44-0.45)
Unmarried 18-19y	0.73 (0.72-0.74)	0.73 (0.72-0.74)	1.00	0.72 (0.71-0.73)	0.72 (0.71-0.73)	1.00	0.74 (0.73-0.75)	0.74 (0.73-0.75)	1.00
Married 18-19y	0.54 (0.53-0.56)	1.20 (1.17-1.22)	0.75 (0.73-0.76)	0.54 (0.52-0.56)	1.14 (1.10-1.18)	0.75 (0.72-0.78)	0.55 (0.53-0.57)	1.24 (1.20-1.29)	0.74 (0.72-0.77)
Unmarried <18y	0.38 (0.38-0.39)	0.38 (0.38-0.39)	1.00	0.39 (0.38-0.40)	0.39 (0.38-0.40)	1.00	0.38 (0.37-0.39)	0.38 (0.37-0.39)	1.00
Married <18y	0.48 (0.44-0.52)	1.05 (0.96-1.13)	1.24 (1.15-1.35)	0.50 (0.45-0.56)	1.05 (0.95-1.18)	1.28 (1.15-1.43)	0.45 (0.40-0.51)	1.02 (0.91-1.15)	1.19 (1.06-1.35)
Late/no prenatal care initiation †	***			***			***		
Unmarried 20-24y	1.00	1.00	1.00	1.00	1.00	1.00	1.00	1.00	1.00
Married 20-24y	0.79 (0.79-0.80)	1.00	0.79 (0.79-0.80)	0.78 (0.78-0.79)	1.00	0.78 (0.78-0.79)	0.80 (0.79-0.80)	1.00	0.80 (0.79-0.80)
Unmarried 18-19y	1.30 (1.29-1.30)	1.30 (1.29-1.30)	1.00	1.28 (1.27-1.29)	1.28 (1.27-1.29)	1.00	1.31 (1.30-1.32)	1.31 (1.30-1.32)	1.00
Married 18-19y	1.21 (1.19-1.22)	1.53 (1.50-1.55)	0.93 (0.92-0.94)	1.20 (1.18-1.22)	1.54 (1.50-1.57)	0.94 (0.92-0.96)	1.21 (1.19-1.23)	1.52 (1.49-1.55)	0.93 (0.91-0.94)
Unmarried <18y	1.80 (1.78-1.81)	1.80 (1.78-1.81)	1.00	1.73 (1.71-1.76)	1.73 (1.71-1.76)	1.00	1.84 (1.82-1.86)	1.84 (1.82-1.86)	1.00
Married <18y	1.58 (1.52-1.64)	1.99 (1.92-2.08)	0.88 (0.84-0.91)	1.61 (1.52-1.70)	2.06 (1.94-2.17)	0.93 (0.88-0.98)	1.55 (1.47-1.64)	1.95 (1.84-2.06)	0.84 (0.80-0.89)

† Adjusted for maternal race/ethnicity, US-born status, parity, paternal age, WIC received, Medicaid as main payor of the delivery, and birth year.
‡ Adjusted for maternal race/ethnicity, US-born status, paternal age, WIC received, Medicaid as main payor of the delivery, and birth year.
§ Adjusted for the same covariates as the primary analysis.
* $p < 0.05$, ** $p < 0.01$, *** $p < 0.001$ for interaction term between marital status and maternal age group.

Maternal health indicator	Adjusted odds ratios (95%CI), primary analysis			Adjusted odds ratios (95%CI) ¶, sensitivity analysis for births recorded in 2014-2015			Adjusted odds ratios (95%CI) ¶, sensitivity analysis for births recorded in 2016-2019		
Marital status & maternal age group	Joint with 1 reference category	By maternal age group within marital status	By marital status within maternal age group	Joint with 1 reference category	By maternal age group within marital status	By marital status within maternal age group	Joint with 1 reference category	By maternal age group within marital status	By marital status within maternal age group
Sexually transmitted infection (STI) †	***			***			***		
Unmarried 20-24y	1.00	1.00	1.00	1.00	1.00	1.00	1.00	1.00	1.00
Married 20-24y	0.45 (0.44-0.45)	1.00	0.45 (0.44-0.45)	0.47 (0.46-0.48)	1.00	0.47 (0.46-0.48)	0.44 (0.43-0.44)	1.00	0.44 (0.43-0.44)
Unmarried 18-19y	1.28 (1.26-1.29)	1.28 (1.26-1.29)	1.00	1.26 (1.24-1.28)	1.26 (1.24-1.28)	1.00	1.28 (1.27-1.30)	1.28 (1.27-1.30)	1.00
Married 18-19y	0.73 (0.71-0.76)	1.64 (1.59-1.70)	0.58 (0.56-0.60)	0.72 (0.69-0.76)	1.55 (1.47-1.64)	0.57 (0.54-0.61)	0.74 (0.71-0.77)	1.70 (1.63-1.77)	0.58 (0.55-0.60)
Unmarried <18y	1.29 (1.27-1.31)	1.29 (1.27-1.31)	1.00	1.29 (1.26-1.32)	1.29 (1.26-1.32)	1.00	1.29 (1.26-1.31)	1.29 (1.26-1.31)	1.00
Married <18y	0.81 (0.74-0.90)	1.82 (1.65-2.01)	0.63 (0.57-0.70)	0.76 (0.65-0.88)	1.63 (1.40-1.90)	0.59 (0.51-0.69)	0.87 (0.76-0.99)	1.99 (1.74-2.26)	0.68 (0.59-0.77)
Gestational hypertension ‡									
Unmarried 20-24y	1.00	1.00	1.00	1.00	1.00	1.00	1.00	1.00	1.00
Married 20-24y	1.02 (1.01-1.03)	1.00	1.02 (1.01-1.03)	1.04 (1.03-1.06)	1.00	1.04 (1.03-1.06)	1.01 (1.00-1.03)	1.00	1.01 (1.00-1.03)
Unmarried 18-19y	0.94 (0.93-0.95)	0.94 (0.93-0.95)	1.00	0.95 (0.93-0.97)	0.95 (0.93-0.97)	1.00	0.93 (0.92-0.94)	0.93 (0.92-0.94)	1.00
Married 18-19y	0.99 (0.96-1.01)	0.96 (0.94-0.99)	1.05 (1.03-1.08)	1.01 (0.97-1.05)	0.97 (0.93-1.01)	1.06 (1.01-1.11)	0.98 (0.95-1.01)	0.96 (0.93-0.99)	1.05 (1.02-1.09)
Unmarried <18y	0.89 (0.87-0.90)	0.89 (0.87-0.90)	1.00	0.90 (0.88-0.93)	0.90 (0.88-0.93)	1.00	0.88 (0.86-0.90)	0.88 (0.86-0.90)	1.00
Married <18y	0.89 (0.82-0.96)	0.87 (0.80-0.94)	1.00 (0.92-1.09)	0.92 (0.81-1.04)	0.88 (0.78-1.00)	1.02 (0.90-1.16)	0.87 (0.78-0.97)	0.85 (0.77-0.95)	0.98 (0.88-1.10)
Eclampsia § *	*						*		
Unmarried 20-24y	1.00	1.00	1.00	1.00	1.00	1.00	1.00	1.00	1.00
Married 20-24y	0.99 (0.95-1.03)	1.00	0.99 (0.95-1.03)	1.13 (1.05-1.21)	1.00	1.13 (1.05-1.21)	0.93 (0.88-0.98)	1.00	0.93 (0.88-0.98)
Unmarried 18-19y	1.04 (0.99-1.09)	1.04 (0.99-1.09)	1.00	1.09 (1.00-1.18)	1.09 (1.00-1.18)	1.00	1.01 (0.95-1.08)	1.01 (0.95-1.08)	1.00
Married 18-19y	1.20 (1.08-1.33)	1.21 (1.08-1.35)	1.15 (1.03-1.29)	1.35 (1.14-1.60)	1.20 (1.01-1.43)	1.24 (1.04-1.48)	1.12 (0.97-1.28)	1.21 (1.05-1.39)	1.10 (0.96-1.27)
Unmarried <18y	1.10 (1.02-1.18)	1.10 (1.02-1.18)	1.00	1.15 (1.02-1.29)	1.15 (1.02-1.29)	1.00	1.07 (0.98-1.17)	1.07 (0.98-1.17)	1.00
Married <18y	1.29 (0.94-1.78)	1.30 (0.94-1.80)	1.18 (0.85-1.63)	1.36 (0.84-2.20)	1.21 (0.75-1.96)	1.19 (0.73-1.94)	1.26 (0.82-1.95)	1.37 (0.89-2.11)	1.18 (0.76-1.83)
Maternal morbidity §	***			**			***		
Unmarried 20-24y	1.00	1.00	1.00	1.00	1.00	1.00	1.00	1.00	1.00
Married 20-24y	1.24 (1.21-1.26)	1.00	1.24 (1.21-1.26)	1.23 (1.19-1.27)	1.00	1.23 (1.19-1.27)	1.24 (1.21-1.28)	1.00	1.24 (1.21-1.28)
Unmarried 18-19y	1.04 (1.01-1.06)	1.04 (1.01-1.06)	1.00	1.06 (1.02-1.10)	1.06 (1.02-1.10)	1.00	1.03 (1.00-1.06)	1.03 (1.00-1.06)	1.00
Married 18-19y	1.11 (1.06-1.17)	0.90 (0.85-0.94)	1.07 (1.02-1.13)	1.14 (1.05-1.23)	0.92 (0.85-1.00)	1.08 (0.99-1.17)	1.09 (1.02-1.17)	0.88 (0.82-0.94)	1.07 (1.00 -1.14)
Unmarried <18y	1.12 (1.08-1.16)	1.12 (1.08-1.16)	1.00	1.12 (1.06-1.18)	1.12 (1.06-1.18)	1.00	1.12 (1.07-1.17)	1.12 (1.07-1.17)	1.00
Married <18y	1.09 (0.94-1.27)	0.88 (0.76-1.03)	0.98 (0.84-1.14)	1.12 (0.90-1.39)	0.91 (0.73-1.13)	1.00 (0.80-1.25)	1.08 (0.87-1.33)	0.87 (0.70-1.07)	0.97 (0.78-1.19)

† Adjusted for maternal race/ethnicity, US-born status, parity, paternal age, WIC received, Medicaid as main payor of the delivery, and birth year.

‡ Adjusted for maternal race/ethnicity, US-born status, parity, maternal smoking, prenatal care adequacy, any diabetes (pre-existing or gestational), paternal age, WIC received, Medicaid as main payor of the delivery, and birth year.

§ Adjusted for maternal race/ethnicity, US-born status, parity, maternal smoking, prenatal care adequacy, any diabetes (pre-existing or gestational), pre-existing hypertension, paternal age, WIC received, Medicaid as main payor of the delivery, and birth year.

¶ Adjusted for the same covariates as the primary analysis.

* $p < 0.05$, ** $p < 0.01$, *** $p < 0.001$ for interaction term between marital status and maternal age group.

Infant health indicator	Adjusted odds ratios (95%CI), primary analysis			Adjusted odds ratios (95%CI) ¶, sensitivity analysis for births recorded in 2014-2015			Adjusted odds ratios (95%CI) ¶, sensitivity analysis for births recorded in 2016-2019		
Marital status & maternal age group	Joint with 1 reference category	By maternal age group within marital status	By marital status within maternal age group	Joint with 1 reference category	By maternal age group within marital status	By marital status within maternal age group	Joint with 1 reference category	By maternal age group within marital status	By marital status within maternal age group
Preterm †	***			***			***		
Unmarried 20-24y	1.00	1.00	1.00	1.00	1.00	1.00	1.00	1.00	1.00
Married 20-24y	0.89 (0.88-0.90)	1.00	0.89 (0.88-0.90)	0.89 (0.87-0.90)	1.00	0.89 (0.87-0.90)	0.90 (0.89-0.91)	1.00	0.90 (0.89-0.91)
Unmarried 18-19y	1.13 (1.12-1.14)	1.13 (1.12-1.14)	1.00	1.13 (1.11-1.15)	1.13 (1.11-1.15)	1.00	1.13 (1.12-1.15)	1.13 (1.12-1.15)	1.00
Married 18-19y	1.11 (1.09-1.14)	1.25 (1.22-1.28)	0.98 (0.96-1.01)	1.11 (1.07-1.15)	1.25 (1.21-1.30)	0.98 (0.95-1.02)	1.12 (1.09-1.15)	1.25 (1.21-1.29)	0.99 (0.96-1.02)
Unmarried <18y	1.22 (1.20-1.24)	1.22 (1.20-1.24)	1.00	1.22 (1.20-1.25)	1.22 (1.20-1.25)	1.00	1.22 (1.20-1.24)	1.22 (1.20-1.24)	1.00
Married <18y	1.22 (1.14-1.31)	1.37 (1.28-1.47)	1.00 (0.93-1.07)	1.23 (1.12-1.36)	1.39 (1.26-1.53)	1.01 (0.91-1.11)	1.21 (1.10-1.33)	1.35 (1.23-1.48)	0.99 (0.90-1.09)
Small for gestational age (SGA) ‡	***			**			**		
Unmarried 20-24y	1.00	1.00	1.00	1.00	1.00	1.00	1.00	1.00	1.00
Married 20-24y	0.91 (0.90-0.92)	1.00	0.91 (0.90-0.92)	0.91 (0.90-0.93)	1.00	0.91 (0.90-0.93)	0.90 (0.89-0.91)	1.00	0.90 (0.89-0.91)
Unmarried 18-19y	1.00 (0.99-1.01)	1.00 (0.99-1.01)	1.00	0.98 (0.96-1.00)	0.98 (0.96-1.00)	1.00	1.02 (1.00-1.03)	1.02 (1.00-1.03)	1.00
Married 18-19y	0.96 (0.93-0.98)	1.06 (1.03-1.09)	0.96 (0.93-0.98)	0.94 (0.90-0.99)	1.03 (0.99-1.08)	0.96 (0.92-1.01)	0.97 (0.93-1.00)	1.07 (1.03-1.11)	0.95 (0.92-0.99)
Unmarried <18y	0.95 (0.94-0.97)	0.95 (0.94-0.97)	1.00	0.93 (0.91-0.96)	0.93 (0.91-0.96)	1.00	0.97 (0.94-0.99)	0.97 (0.94-0.99)	1.00
Married <18y	1.01 (0.93-1.09)	1.11 (1.02-1.21)	1.06 (0.97-1.15)	1.02 (0.91-1.14)	1.12 (0.99-1.26)	1.10 (0.97-1.23)	0.99 (0.88-1.12)	1.10 (0.98-1.24)	1.03 (0.91-1.16)
Infant morbidity †	***			***			***		
Unmarried 20-24y	1.00	1.00	1.00	1.00	1.00	1.00	1.00	1.00	1.00
Married 20-24y	0.92 (0.91-0.93)	1.00	0.92 (0.91-0.93)	0.91 (0.90-0.92)	1.00	0.91 (0.90-0.92)	0.93 (0.92-0.94)	1.00	0.93 (0.92-0.94)
Unmarried 18-19y	0.97 (0.96-0.98)	0.97 (0.96-0.98)	1.00	0.96 (0.95-0.98)	0.96 (0.95-0.98)	1.00	0.98 (0.96-0.99)	0.98 (0.96-0.99)	1.00
Married 18-19y	0.95 (0.93-0.97)	1.03 (1.01-1.05)	0.98 (0.96-1.00)	0.94 (0.91-0.97)	1.04 (1.00-1.07)	0.98 (0.94-1.01)	0.95 (0.93-0.98)	1.03 (1.00-1.06)	0.98 (0.95-1.00)
Unmarried <18y	0.94 (0.93-0.96)	0.94 (0.93-0.96)	1.00	0.94 (0.92-0.96)	0.94 (0.92-0.96)	1.00	0.95 (0.93-0.96)	0.95 (0.93-0.96)	1.00
Married <18y	1.01 (0.95-1.08)	1.10 (1.04-1.17)	1.07 (1.01-1.14)	0.95 (0.87-1.04)	1.05 (0.95-1.15)	1.02 (0.92-1.12)	1.07 (0.98-1.16)	1.15 (1.06-1.25)	1.12 (1.03-1.22)
Infant not breastfed at discharge §	***			***			***		
Unmarried 20-24y	1.00	1.00	1.00	1.00	1.00	1.00	1.00	1.00	1.00
Married 20-24y	0.64 (0.63-0.64)	1.00	0.64 (0.63-0.64)	0.65 (0.64-0.65)	1.00	0.65 (0.64-0.65)	0.63 (0.63-0.64)	1.00	0.63 (0.63-0.64)
Unmarried 18-19y	1.24 (1.23-1.25)	1.24 (1.23-1.25)	1.00	1.23 (1.22-1.24)	1.23 (1.22-1.24)	1.00	1.25 (1.24-1.26)	1.25 (1.24-1.26)	1.00
Married 18-19y	0.86 (0.84-0.87)	1.35 (1.32-1.37)	0.69 (0.68-0.70)	0.87 (0.85-0.89)	1.35 (1.31-1.38)	0.71 (0.69-0.73)	0.85 (0.83-0.87)	1.35 (1.32-1.38)	0.68 (0.67-0.70)
Unmarried <18y	1.58 (1.56-1.59)	1.58 (1.56-1.59)	1.00	1.60 (1.58-1.63)	1.60 (1.58-1.63)	1.00	1.57 (1.55-1.59)	1.57 (1.55-1.59)	1.00
Married <18y	1.12 (1.07-1.18)	1.76 (1.67-1.85)	0.71 (0.68-0.75)	1.14 (1.06-1.22)	1.76 (1.64-1.89)	0.71 (0.66-0.76)	1.11 (1.04-1.19)	1.76 (1.65-1.89)	0.71 (0.66-0.76)

† Adjusted for infant sex, maternal race/ethnicity, US-born status, parity, maternal smoking, prenatal care adequacy, any diabetes (pre-existing or gestational), pre-existing hypertension, paternal age, WIC received, Medicaid as main payor of the delivery, and birth year.

‡ Adjusted for maternal race/ethnicity, US-born status, parity, maternal smoking, prenatal care adequacy, any diabetes (pre-existing or gestational), pre-existing hypertension, paternal age, WIC received, Medicaid as main payor of the delivery, and birth year.

§ Adjusted for maternal race/ethnicity, US-born status, parity, maternal smoking, prenatal care adequacy, paternal age, WIC received, Medicaid as main payor of the delivery, and birth year.

¶ Adjusted for the same covariates as the primary analysis.

* $p < 0.05$, ** $p < 0.01$, *** $p < 0.001$ for interaction term between marital status and maternal age group.
