# Supplementary material for: Reproductive health among married and unmarried mothers aged less than 18, 18–19, and 20–24 years in the United States, 2014–2019: A population-based cross-sectional study
Source: PLoS Med. 2022 Mar 10;19(3):e1003929. doi: 10.1371/journal.pmed.1003929 (PMC8912259; doi:10.1371/journal.pmed.1003929)
Supplement: S6 File — (PDF) [file pmed.1003929.s008.pdf]

S6 File. Adjusted odds ratios of maternal and infant health indicators associated with the interaction between marital status and maternal age group, sensitivity analysis with maternal pre-pregnancy body mass index (BMI)

**Maternal health indicator**	**Adjusted odds ratios (95%CI), primary analysis**			**Adjusted odds ratios (95%CI) ¶, sensitivity analysis with pre-pregnancy BMI**		
Marital status & maternal age group	Joint with 1 reference category	By maternal age group within marital status	By marital status within maternal age group	Joint with 1 reference category	By maternal age group within marital status	By marital status within maternal age group
Gestational hypertension ‡						
Unmarried 20-24y	1.00	1.00	1.00	1.00	1.00	1.00
Married 20-24y	1.02 (1.01-1.03)	1.00	1.02 (1.01-1.03)	1.01 (1.00-1.02)	1.00	1.01 (1.00-1.02)
Unmarried 18-19y	0.94 (0.93-0.95)	0.94 (0.93-0.95)	1.00	0.99 (0.98-1.01)	0.99 (0.98-1.01)	1.00
Married 18-19y	0.99 (0.96-1.01)	0.96 (0.94-0.99)	1.05 (1.03-1.08)	1.03 (1.00-1.05)	1.01 (0.99-1.04)	1.03 (1.00-1.06)
Unmarried <18y	0.89 (0.87-0.90)	0.89 (0.87-0.90)	1.00	1.01 (0.99-1.02)	1.01 (0.99-1.02)	1.00
Married <18y	0.89 (0.82-0.96)	0.87 (0.80-0.94)	1.00 (0.92-1.09)	0.97 (0.89-1.06)	0.96 (0.88-1.04)	0.97 (0.89-1.05)
Eclampsia § *	*			*		
Unmarried 20-24y	1.00	1.00	1.00	1.00	1.00	1.00
Married 20-24y	0.99 (0.95-1.03)	1.00	0.99 (0.95-1.03)	0.98 (0.94-1.03)	1.00	0.98 (0.94-1.03)
Unmarried 18-19y	1.04 (0.99-1.09)	1.04 (0.99-1.09)	1.00	1.08 (1.03-1.13)	1.08 (1.03-1.13)	1.00
Married 18-19y	1.20 (1.08-1.33)	1.21 (1.08-1.35)	1.15 (1.03-1.29)	1.23 (1.10-1.37)	1.25 (1.12-1.39)	1.14 (1.02-1.27)
Unmarried <18y	1.10 (1.02-1.18)	1.10 (1.02-1.18)	1.00	1.19 (1.10-1.27)	1.19 (1.10-1.27)	1.00
Married <18y	1.29 (0.94-1.78)	1.30 (0.94-1.80)	1.18 (0.85-1.63)	1.37 (0.99-1.88)	1.39 (1.00-1.91)	1.15 (0.83-1.60)
Maternal morbidity §	***			***		
Unmarried 20-24y	1.00	1.00	1.00	1.00	1.00	1.00
Married 20-24y	1.24 (1.21-1.26)	1.00	1.24 (1.21-1.26)	1.24 (1.22-1.27)	1.00	1.24 (1.22-1.27)
Unmarried 18-19y	1.04 (1.01-1.06)	1.04 (1.01-1.06)	1.00	1.02 (1.00-1.05)	1.02 (1.00-1.05)	1.00
Married 18-19y	1.11 (1.06-1.17)	0.90 (0.85-0.94)	1.07 (1.02-1.13)	1.10 (1.05-1.16)	0.89 (0.84-0.93)	1.08 (1.02-1.13)
Unmarried <18y	1.12 (1.08-1.16)	1.12 (1.08-1.16)	1.00	1.08 (1.05-1.12)	1.08 (1.05-1.12)	1.00
Married <18y	1.09 (0.94-1.27)	0.88 (0.76-1.03)	0.98 (0.84-1.14)	1.07 (0.92-1.24)	0.86 (0.74-1.00)	0.99 (0.85-1.15)

† Adjusted for maternal race/ethnicity, US-born status, parity, paternal age, WIC received, Medicaid as main payor of the delivery, and birth year.

‡ Adjusted for maternal race/ethnicity, US-born status, parity, maternal smoking, prenatal care adequacy, any diabetes (pre-existing or gestational), paternal age, WIC received, Medicaid as main payor of the delivery, and birth year.

§ Adjusted for maternal race/ethnicity, US-born status, parity, maternal smoking, prenatal care adequacy, any diabetes (pre-existing or gestational), pre-existing hypertension, paternal age, WIC received, Medicaid as main payor of the delivery, and birth year.

¶ Adjusted for the same covariates as the primary analysis and for pre-pregnancy body mass index (BMI).

* $p < 0.05$, ** $p < 0.01$, *** $p < 0.001$ for interaction term between marital status and maternal age group.

Infant health indicator	Adjusted odds ratios (95%CI), primary analysis			Adjusted odds ratios (95%CI) ¶, sensitivity analysis with pre-pregnancy BMI		
Marital status & maternal age group	Joint with 1 reference category	By maternal age group within marital status	By marital status within maternal age group	Joint with 1 reference category	By maternal age group within marital status	By marital status within maternal age group
Preterm †	***			***		
Unmarried 20-24y	1.00	1.00	1.00	1.00	1.00	1.00
Married 20-24y	0.89 (0.88-0.90)	1.00	0.89 (0.88-0.90)	0.90 (0.89-0.90)	1.00	0.90 (0.89-0.90)
Unmarried 18-19y	1.13 (1.12-1.14)	1.13 (1.12-1.14)	1.00	1.12 (1.11-1.13)	1.12 (1.11-1.13)	1.00
Married 18-19y	1.11 (1.09-1.14)	1.25 (1.22-1.28)	0.98 (0.96-1.01)	1.11 (1.08-1.13)	1.24 (1.21-1.26)	0.99 (0.97-1.01)
Unmarried <18y	1.22 (1.20-1.24)	1.22 (1.20-1.24)	1.00	1.20 (1.18-1.21)	1.20 (1.18-1.21)	1.00
Married <18y	1.22 (1.14-1.31)	1.37 (1.28-1.47)	1.00 (0.93-1.07)	1.20 (1.12-1.28)	1.34 (1.25-1.43)	1.00 (0.94-1.07)
Small for gestational age (SGA) ‡	***			***		
Unmarried 20-24y	1.00	1.00	1.00	1.00	1.00	1.00
Married 20-24y	0.91 (0.90-0.92)	1.00	0.91 (0.90-0.92)	0.91 (0.90-0.92)	1.00	0.91 (0.90-0.92)
Unmarried 18-19y	1.00 (0.99-1.01)	1.00 (0.99-1.01)	1.00	0.98 (0.97-0.99)	0.98 (0.97-0.99)	1.00
Married 18-19y	0.96 (0.93-0.98)	1.06 (1.03-1.09)	0.96 (0.93-0.98)	0.94 (0.91-0.97)	1.03 (1.00-1.06)	0.96 (0.94-0.99)
Unmarried <18y	0.95 (0.94-0.97)	0.95 (0.94-0.97)	1.00	0.91 (0.89-0.92)	0.91 (0.89-0.92)	1.00
Married <18y	1.01 (0.93-1.09)	1.11 (1.02-1.21)	1.06 (0.97-1.15)	0.97 (0.89-1.05)	1.06 (0.98-1.15)	1.07 (0.98-1.16)
Infant morbidity †	***			***		
Unmarried 20-24y	1.00	1.00	1.00	1.00	1.00	1.00
Married 20-24y	0.92 (0.91-0.93)	1.00	0.92 (0.91-0.93)	0.92 (0.91-0.93)	1.00	0.92 (0.91-0.93)
Unmarried 18-19y	0.97 (0.96-0.98)	0.97 (0.96-0.98)	1.00	0.98 (0.97-0.98)	0.98 (0.97-0.98)	1.00
Married 18-19y	0.95 (0.93-0.97)	1.03 (1.01-1.05)	0.98 (0.96-1.00)	0.95 (0.93-0.97)	1.03 (1.01-1.06)	0.97 (0.95-1.00)
Unmarried <18y	0.94 (0.93-0.96)	0.94 (0.93-0.96)	1.00	0.95 (0.94-0.96)	0.95 (0.94-0.96)	1.00
Married <18y	1.01 (0.95-1.08)	1.10 (1.04-1.17)	1.07 (1.01-1.14)	1.02 (0.95-1.08)	1.11 (1.04-1.18)	1.07 (1.00-1.14)

† Adjusted for infant sex, maternal race/ethnicity, US-born status, parity, maternal smoking, prenatal care adequacy, any diabetes (pre-existing or gestational), pre-existing hypertension, paternal age, WIC received, Medicaid as main payor of the delivery, and birth year.

‡ Adjusted for maternal race/ethnicity, US-born status, parity, maternal smoking, prenatal care adequacy, any diabetes (pre-existing or gestational), pre-existing hypertension, paternal age, WIC received, Medicaid as main payor of the delivery, and birth year.

§ Adjusted for maternal race/ethnicity, US-born status, parity, maternal smoking, prenatal care adequacy, paternal age, WIC received, Medicaid as main payor of the delivery, and birth year.

¶ Adjusted for the same covariates as the primary analysis and for pre-pregnancy body mass index (BMI).

* $p < 0.05$, ** $p < 0.01$, *** $p < 0.001$ for interaction term between marital status and maternal age group.
