# Supplementary material for: Reproductive health among married and unmarried mothers aged less than 18, 18–19, and 20–24 years in the United States, 2014–2019: A population-based cross-sectional study
Source: PLoS Med. 2022 Mar 10;19(3):e1003929. doi: 10.1371/journal.pmed.1003929 (PMC8912259; doi:10.1371/journal.pmed.1003929)
Supplement: S1 Table — (PDF) [file pmed.1003929.s011.pdf]

S1 Table. Characteristics of births to US mothers aged ≤24 years* by marital status availability

Characteristics	Available marital status (N = 5,669,824) n (%)	Missing marital status (N = 269,341) n (%)	|*d*|
Maternal age			
<18 years	314,098 (5.5)	13,239 (4.9)	0.03
18-19 years	873,111 (15.4)	38,980 (14.5)	0.03
20-24 years	4,482,615 (79.1)	217,122 (80.6)	0.04
Maternal race/ethnicity			
White	2,552,211 (45.0)	43,940 (16.3)	0.65
Black	1,146,440 (20.2)	18,336 (6.8)	0.40
American Indian & Alaska Natives	70,432 (1.2)	1275 (0.5)	0.08
Asian	101,023 (1.8)	9509 (3.5)	0.11
Native Hawaiian & Pacific Islander	16,390 (0.3)	1180 (0.4)	0.02
Multi-race	169,506 (3.0)	7091 (2.6)	0.02
Hispanic †	1,584,981 (28.0)	181,316 (67.3)	0.86
Not available	28,841 (0.5)	6694 (2.5)	0.16
Maternal nativity status			
US-born	4,872,887 (85.9)	208,699 (77.5)	0.22
Foreign-born	784,746 (13.8)	59,968 (22.3)	0.22
Not available	12,191 (0.2)	674 (0.3)	0.01
Paternal age			
<18 y	76,927 (1.4)	4443 (1.7)	0.02
18-19 y	317,208 (5.6)	16,971 (6.3)	0.03
20-24 y	2,102,628 (37.1)	110,499 (41.0)	0.08
≥25 y	1,917,517 (33.8)	100,327 (37.3)	0.07
Not available	1,255,544 (22.1)	37,101 (13.8)	0.22
WIC received			
No	2,133,762 (37.6)	80,963 (30.1)	0.16
Yes	3,447,877 (60.8)	185,890 (69.0)	0.17
Not available	88,185 (1.6)	2488 (0.9)	0.06
Medicaid as main payor			
No	1,890,942 (33.4)	87,414 (32.5)	0.02
Yes	3,735,836 (65.9)	181,262 (67.3)	0.03
Not available	43,046 (0.8)	665 (0.3)	0.07
Birth year			
2014	1,105,798 (19.5)	787 (0.3)	0.68
2015	1,069,474 (18.9)	465 (0.2)	0.67
2016	1,015,587 (17.9)	438 (0.2)	0.65
2017	864,105 (15.2)	96,952 (36.0)	0.49
2018	819,686 (14.5)	88,084 (32.7)	0.44
2019	795,174 (14.0)	82,615 (30.7)	0.41
Infant sex			
Male	2,902,188 (51.2)	137,659 (51.1)	0.00
Female	2,767,636 (48.8)	131,682 (48.9)	0.00
Any maternal smoking			
No	5,019,455 (88.5)	261,360 (97.0)	0.33
Yes	588,820 (10.4)	4659 (1.7)	0.37
Not available	61,549 (1.1)	3322 (1.2)	0.01
Parity			
0	3,297,019 (58.2)	164,290 (61.0)	0.06
1	1,620,737 (28.6)	75,231 (27.9)	0.01
≥2	732,143 (12.9)	29,385 (10.9)	0.06
Not available	19,925 (0.4)	435 (0.2)	0.04
Any diabetes ‡			
No	5,457,603 (96.3)	260,273 (96.6)	0.02
Yes	205,177 (3.6)	8975 (3.3)	0.02
Not available	7044 (0.1)	93 (0.03)	0.03
Pre-existing hypertension			
No	5,605,045 (98.9)	267,863 (99.5)	0.06
Yes	57,735 (1.0)	1385 (0.5)	0.06

Not available	7044 (0.1)	93 (0.03)	0.03
Prenatal care adequacy §			
No care	132,032 (2.3)	3987 (1.5)	0.06
Intensive	363,329 (6.4)	19,907 (7.4)	0.04
Adequate	1,990,794 (35.1)	101,548 (37.7)	0.05
Intermediate	2,292,151 (40.4)	113,585 (42.2)	0.04
Inadequate	704,581 (12.4)	25,742 (9.6)	0.09
Not available	186,937 (3.3)	4572 (1.7)	0.10
Maternal education #			
≤8th grade	173,696 (3.1)	8646 (3.2)	0.01
9-12th grade, no diploma	1,169,295 (20.6)	45,389 (16.9)	0.10
High school graduate / GED	2,396,840 (42.3)	117,405 (43.6)	0.03
Above high school / GED	1,881,603 (33.2)	84,918 (31.5)	0.04
Not available	48,390 (0.9)	12,983 (4.8)	0.24
Maternal pre-pregnancy BMI			
Underweight (<18.5)	299,514 (5.3)	12,436 (4.6)	0.03
Normal weight (18.5-24.9)	2,455,274 (43.3)	111,075 (41.2)	0.04
Overweight (25.0-29.9)	1,359,275 (24.0)	70,978 (26.4)	0.05
Obesity (≥30)	1,396,193 (24.6)	67,205 (25.0)	0.01
Not available	159,568 (2.8)	7647 (2.8)	0.00
Paternal education #			
≤8th grade	174,450 (3.1)	11,040 (4.1)	0.05
9-12th grade, no diploma	785,594 (13.9)	38,834 (14.4)	0.02
High school graduate or GED	2,011,438 (35.5)	108,681 (40.4)	0.10
Above high school / GED	1,311,038 (23.1)	60,892 (22.6)	0.01
Not available	1,387,304 (24.5)	49,894 (18.5)	0.15
Parental age gap			
Mother older than father	466,971 (8.2)	25,883 (9.6)	0.05
Father 0-2 years older	1,983,832 (35.0)	106,649 (39.6)	0.10
Father 3-4 years older	839,662 (14.8)	41,069 (15.3)	0.01
Father 5-9 years older	792,939 (14.0)	40,911 (15.2)	0.03
Father ≥10 years older	330,876 (5.8)	17,728 (6.6)	0.03
Not available	1,255,544 (22.1)	37,101 (13.8)	0.22

Column percent may not add to 100 because of rounding.

|*d*| absolute standardized difference [1] between births to married and unmarried mothers within maternal age group. A standardized difference of ≤0.1 indicates negligeable difference between the two groups [1].

* Includes all births to US mothers aged ≤24 years with data recorded using the 2003 US Standard Certificate of Live Birth.

† Includes all mothers who self-identified as Hispanic with or without another race/ethnicity.

‡ Pre-existing or gestational diabetes.

§ Derived using the revised Graduated Prenatal Care Utilization Index (GINDEX) [2].

# When the level of maternal educational attainment is not compatible with maternal age (i.e., educational level too high for maternal age), the education level was edited as "Not available" as per the consistency checks applied by the Division of Vital Statistics in the 2018 and 2019 Natality Public Use Files [3, 4]. The same consistency checks were applied to paternal education based on paternal age for all years.

References

1. Austin PC. Using the Standardized Difference to Compare the Prevalence of a Binary Variable Between Two Groups in Observational Research. Commun Stat Simul Comput. 2009;38(6):1228-1234.
2. Alexander GR, Kotelchuck M. Quantifying the adequacy of prenatal care: a comparison of indices. Public Health Rep 1996;111(5):408-419.
3. National Center for Health Statistics. User guide to the 2018 Natality Public Use File. Hyattsville, MD: Centers for Disease Control and Prevention; 2019.
4. National Center for Health Statistics. User guide to the 2019 Natality Public Use File. Hyattsville, MD: Centers for Disease Control and Prevention; 2020.
